# Therapeutic Potential of 2-(2-Benzofuranyl)-2-Imidazoline in Preclinical CNS Models: A Systematic Review of Mechanisms, Disease Models, and Cellular Targets

**DOI:** 10.3390/ph19081155

**Published:** 2026-07-24

**Authors:** In-Ae Choi, Ji Hee Yun, Jongmin Lee, Dong-Hee Choi

**Affiliations:** 1 Department of Occupational Therapy, Division of Health, Baekseok University, Cheonan-si 31065, Republic of Korea; inaes2@bu.ac.kr; 2 Department of Rehabilitation Medicine, Konkuk University School of Medicine and Konkuk University Medical Center, Seoul 03080, Republic of Korea; yungane1@naver.com (J.H.Y.); leej@kuh.ac.kr (J.L.); 3 Department of Medical Science, Konkuk University School of Medicine, Konkuk University, Chungju 27478, Republic of Korea

**Keywords:** 2-BFI, imidazoline I_2_-site ligand, neuroprotection, preclinical CNS models, NMDAR-mediated excitotoxicity, oxidative stress, neuroinflammation, blood–brain barrier, neurovascular unit

## Abstract

**Background/Objectives:** 2-(2-Benzofuranyl)-2-imidazoline (2-BFI) is a selective imidazoline I_2_-site ligand that has shown neuroprotective and neuromodulatory effects in preclinical central nervous system (CNS) studies. However, the primary preclinical literature remains fragmented across disease models, outcome types, mechanistic endpoints, and cellular targets, making it difficult to define where its therapeutic-development potential is strongest and how disease- or model-specific functional effects relate to molecular, cellular, tissue, and blood–brain barrier/neurovascular unit (BBB/NVU)-related findings. **Methods:** This systematic review integrated preclinical evidence from 36 original studies identified in the PubMed, Web of Science, Embase, and Scopus databases through searches last updated on May 19, 2026, to evaluate the strength of evidence for 2-BFI across CNS-related models and to connect functional, molecular, cellular, and neurovascular findings. The evidence categories included ischemic stroke/neurovascular outcomes (n = 13), traumatic CNS injury (n = 2), neuroinflammatory/neurodegeneration-related models (n = 9), behavioral pharmacology (n = 8), and cellular mechanisms (n = 4). Eligible studies were original CNS-related animal, cellular, or behavioral/pharmacological studies that directly evaluated 2-BFI and reported neuroprotective, neurological, cellular, molecular, vascular, inflammatory, neurotransmitter-related, or behavioral outcomes. Findings were synthesized qualitatively, and risk of bias in in vivo animal studies was assessed using SYRCLE’s risk-of-bias tool. **Results:** The most extensive preclinical evidence was found in ischemic stroke and neurovascular injury models, in which 2-BFI attenuated infarct size, neurological deficits, and edema, and suppressed apoptosis-related injury and blood–brain barrier/neurovascular unit (BBB/NVU) disruption. Across models, these effects are best interpreted as modulation of interconnected secondary injury processes involving N-methyl-D-aspartate receptor (NMDAR)/Ca^2+^-dependent excitotoxicity, oxidative and mitochondrial stress, inflammatory amplification, regulated cell death, and neurovascular destabilization. Evidence from traumatic CNS injury, autoimmune neuroinflammation, Alzheimer’s disease-related models, chronic epilepsy, and cellular stress models broadened the CNS relevance of 2-BFI but remained less replicated or more mechanistically indirect than the stroke/neurovascular evidence. Behavioral and pharmacological studies additionally indicated that 2-BFI modulates neurotransmitter-related systems associated with pain-, affective-, addiction-, opioid-, and compulsivity-related outcomes, although these findings should be distinguished from disease-modifying neuroprotective evidence. **Conclusions:** Meta-analysis was not conducted because of heterogeneity in models, dosing regimens, treatment timing, and outcomes. Overall, the current evidence does not yet support definitive dosing, treatment timing, or clinical development recommendations for 2-BFI. The strongest preclinical therapeutic rationale is currently found in ischemic stroke and neurovascular injury settings, whereas other CNS indications require further validation. Future studies should define dose–response relationships, therapeutic windows, pharmacokinetic and safety profiles, sex- and age-related effects, and efficacy in clinically relevant comorbid models before clinical translation is considered. The review was not prospectively registered. Funding was provided by a National Research Foundation of Korea grant funded by the Korean government.

## 1. Introduction

Central nervous system (CNS) disorders, including stroke, Alzheimer’s disease (AD), multiple sclerosis (MS), traumatic brain injury (TBI), and various other neuropsychiatric conditions, remain leading causes of disability and mortality worldwide [1]. Despite advances in acute care, many CNS disorders still lack broadly effective treatments that modify underlying pathological processes, rather than only providing symptomatic relief, partly because of their multifactorial pathophysiology involving excitotoxicity, oxidative stress, neuroinflammation, and blood–brain barrier (BBB) disruption [2,3]. This complexity has encouraged the preclinical evaluation of various compounds capable of modulating multiple injury-related pathways.

Among various emerging candidates, imidazoline I_2_ binding sites have attracted attention as CNS-relevant modulatory targets. Initially identified through high-affinity binding to imidazoline-related ligands, I_2_ binding sites have since been implicated in mitochondrial regulation, glial activation, neuroinflammation, and pain modulation [4,5,6]. Their association with mitochondrial compartments and astrocyte-related CNS processes provides a pharmacological basis for the investigation of I_2_-site ligands in redox regulation, neuroinflammation, and cell-death-related pathways [4,5,6]. Because mitochondrial dysfunction, astrocyte-mediated metabolic and inflammatory responses, and oxidative stress are shared contributors to secondary injury in ischemic, traumatic, and neurodegeneration-related CNS disorders, these associations are pharmacologically relevant for evaluating I_2_-site ligands in preclinical CNS models.

2-(2-benzofuranyl)-2-imidazoline (2-BFI), a prototypical selective I_2_-site ligand, has been evaluated in multiple preclinical CNS models, including ischemic stroke [7,8,9,10,11,12,13,14,15,16], traumatic CNS injury [17,18], autoimmune neuroinflammation [19,20,21,22], neurodegeneration-related models [23,24,25,26,27,28], and behavioral or pharmacological models [24,29,30,31,32,33,34,35,36]. This broad experimental use makes 2-BFI a useful ligand for examining whether I_2_-site modulation is associated with convergent neuroprotective, neurovascular, inflammatory, and behavioral effects across CNS-related models. 2-BFI has additionally been used as a pharmacological reference ligand in neuroimaging studies targeting I_2_ binding sites, particularly for assessing the binding specificity of newly developed positron emission tomography (PET) radiotracers [37].

A systematic synthesis of 2-BFI-related preclinical evidence across CNS disease models, mechanisms, and cellular and molecular targets remains lacking. More importantly, it remains unclear which CNS models provide the strongest therapeutic rationale for 2-BFI, how differences in experimental model, dosing regimen, treatment timing, and outcome measures influence interpretation, and how molecular, cellular, neurovascular, and functional findings are connected across studies. Therefore, this review systematically examines the available preclinical evidence for 2-BFI not only to summarize reported effects, but also to evaluate the relative strength of evidence across disease models, identify convergent mechanisms, distinguish disease-modifying neuroprotective evidence from broader neuromodulatory pharmacology, and define translational gaps that should be addressed before further therapeutic development. In this review, 2-BFI is therefore interpreted as a preclinical neuroprotective and neuromodulatory ligand, rather than as an established disease-modifying therapeutic agent. Evidence with potential disease-modifying relevance is considered only when functional or behavioral improvement is linked to tissue, cellular, molecular, inflammatory, or neurovascular protection.

## 2. Methods

### 2.1. Reporting Guideline and Protocol Registration

This systematic review was reported in accordance with the Preferred Reporting Items for Systematic Reviews and Meta-Analyses 2020 (PRISMA 2020) [38] statement. The review protocol was not prospectively registered, and no separate protocol was prepared. Therefore, protocol amendments were not applicable.

### 2.2. Literature Search Strategy

A systematic literature search using the PubMed, Web of Science, Embase, and Scopus databases was conducted to identify studies evaluating 2-(2-benzofuranyl)-2-imidazoline (2-BFI) in CNS-related experimental models. The search used two main query sets: 2-BFI AND (brain OR neuro*) and 2-BFI AND (inhibit OR protect OR neuroprotect), with database-specific syntax adapted for each source. Because the search strategy was centered on 2-BFI combined with broad CNS- and protection-related terms, relevant studies using only behavioral or cognitive terminology could theoretically have been missed. The final search was updated on 19 May 2026.

Before duplicate removal, the search identified 139 records from PubMed (n = 58), Web of Science (n = 39), Embase (n = 10), and Scopus (n = 32). The complete database-specific search strings, search steps, and numbers of records retrieved from each database are provided in Supplementary Table S1.

### 2.3. Eligibility Criteria

Original experimental studies were deemed eligible if they directly evaluated 2-BFI in CNS-related animal models, primary CNS cell systems, cellular stress models, or behavioral/pharmacological models and reported neuroprotective, neurological, cellular, molecular, vascular, inflammatory, neurotransmitter-related, or behavioral outcomes. Studies were excluded if they were reviews, background articles, binding or radioligand studies without eligible experimental outcomes, non-CNS or peripheral pharmacology studies, or studies in which 2-BFI was not directly tested as an active compound. Binding, radioligand, receptor-characterization, or PET tracer studies that did not report eligible CNS disease-, injury-, cellular-, or behavioral-outcome data were excluded from the qualitative synthesis but could be cited as background evidence for the pharmacological identity or I_2_-site selectivity of 2-BFI.

Studies were grouped for qualitative synthesis according to the preclinical disease or behavioral model, primary mechanistic target, and major outcome category.

### 2.4. Study Selection

**Figure 1:**
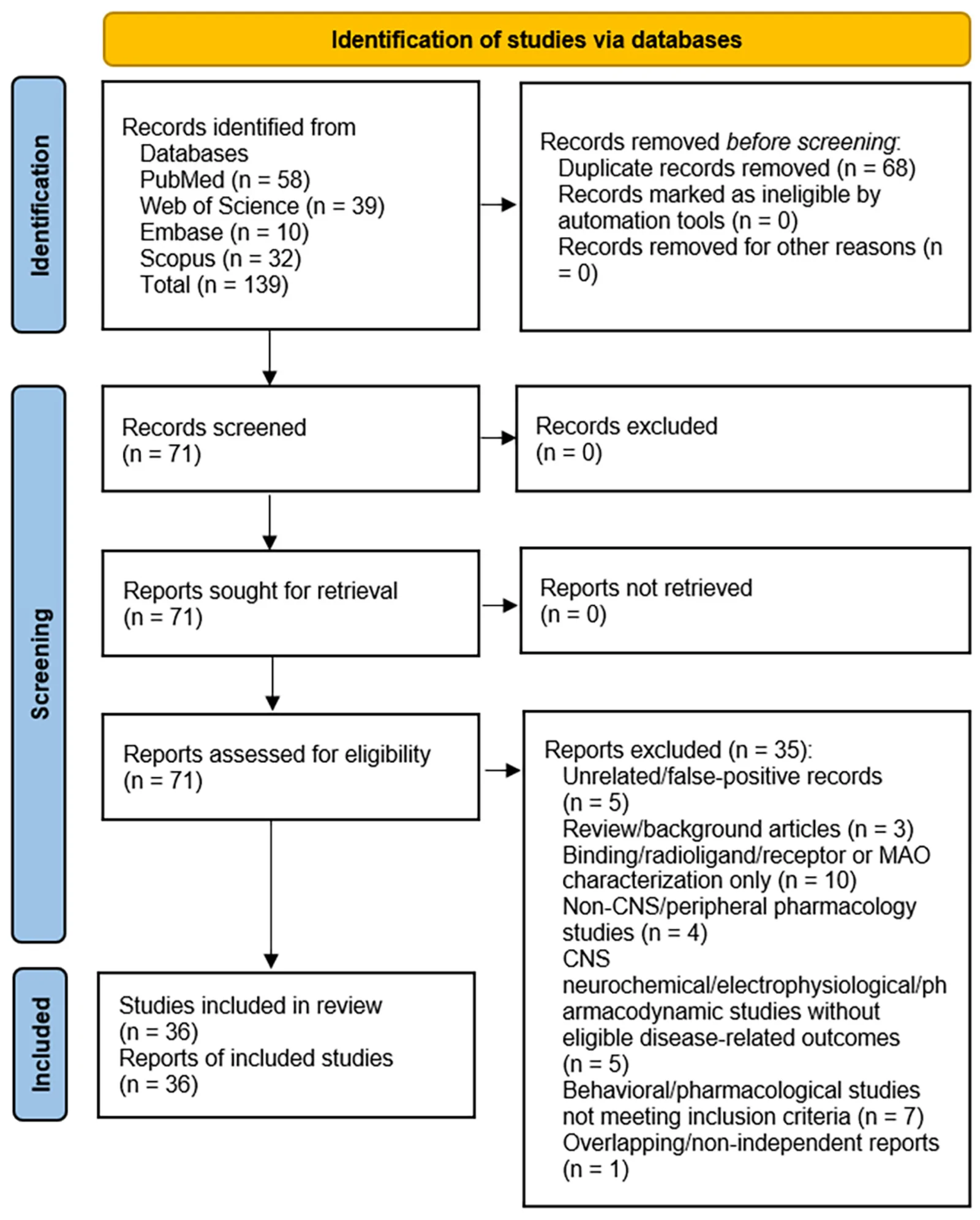
PRISMA 2020 flow diagram showing the identification, screening, eligibility assessment, and inclusion of studies. Detailed exclusion categories and record-level exclusion reasons are provided in Supplementary Tables S2A and S2B.

After excluding 68 duplicate records, 71 unique records were screened and assessed for eligibility. Two reviewers (I.A.C. and D.H.C.) independently evaluated the titles, abstracts, and full texts according to predefined criteria. Disagreements were resolved by consensus, with consultations from J.H.Y. and J.L. if needed. All reports were available for retrieval, and no reports were classified as not retrieved. No automation tools were used for study selection. Following eligibility assessment, 35 reports were excluded, and 36 studies were included in the qualitative synthesis. The study selection process is summarized in the PRISMA 2020 flow diagram in Figure 1.

### 2.5. Exclusion Categories

The 35 excluded reports were assigned a single primary reason for exclusion. Exclusion categories were as follows: unrelated or false-positive records (n = 5), review/background articles without eligible original data (n = 3), binding/radioligand/receptor or monoamine oxidase (MAO)-related characterization only (n = 10), non-CNS or peripheral pharmacology studies (n = 4), CNS neurochemical/electrophysiological/pharmacodynamic studies without assessment of any eligible disease-related protective or functional outcomes (n = 5), behavioral/pharmacological studies not meeting the predefined inclusion criteria (n = 7), and overlapping or non-independent reports not counted as separate studies (n = 1). The excluded reports are summarized by the primary exclusion category in Supplementary Table S2A, and the full record-level exclusion list is provided in Supplementary Table S2B.

### 2.6. Data Extraction and Evidence Classification

For each included study, data on author and year, evidence classification, primary target, experimental model, species, strain, sex, age or weight when reported, cell type when applicable, disease or behavioral model, 2-BFI dose and route, treatment timing, evaluation time point, functional outcomes, tissue or histological outcomes, cellular or molecular outcomes, reported 2-BFI-associated effects, and proposed mechanisms were extracted. Outcomes of interest included neuroprotective, neurological, behavioral, histological, cellular, molecular, vascular, inflammatory, neurotransmitter-related, and mechanistic outcomes related to CNS injury, disease, or behavioral phenotypes. When information was unclear or not reported in the original study, it was recorded as not reported, and no assumptions were made to infer missing data. Data extraction was performed independently by two reviewers (I.A.C. and D.H.C.), and discrepancies were resolved by consensus, with consultation from J.H.Y. and J.L. if needed. No automation tools were used for data extraction.

The included studies were classified by evidence type, primary targets, experimental model, and key outcomes. Evidence classifications included ischemic stroke and neurovascular outcomes, traumatic CNS injury, neuroinflammatory and neurodegenerative models, neuropsychiatric, pain-, and addiction-related phenotypes, and cellular or organelle-focused mechanisms. Mechanism-related findings were further summarized in relation to NMDA receptor-mediated excitotoxicity, oxidative and mitochondrial stress, neuroinflammation and immune modulation, regulated cell death, BBB/neurovascular unit (NVU) integrity, lysosomal/autophagy-related responses, and neurotransmitter-related modulation.

### 2.7. Study Risk-of-Bias Assessment

The risk of bias for in vivo animal studies was assessed using SYRCLE’s risk-of-bias tool. Two reviewers (I.A.C. and D.H.C.) independently evaluated each applicable study as having a low, high, or unclear risk of bias for each item, and disagreements were resolved by consensus. In vitro-only studies were marked as not applicable. No overall risk-of-bias score was calculated. No automation tools were used for risk-of-bias assessment. Study-level judgments and item-level summaries are provided in Supplementary Tables S3A and S3B, respectively. Among the 36 included studies, 31 in vivo or in vivo-containing studies were applicable to SYRCLE’s risk-of-bias assessment, whereas five in vitro-only studies were marked as not applicable. Across the applicable studies, unclear risk due to insufficient methodological reporting predominated in sequence generation (30/31), baseline characteristics (27/31), allocation concealment (31/31), random housing (31/31), blinding of caregivers or investigators (27/31), random outcome assessment (27/31), and other sources of bias (31/31). Outcome-assessor blinding was judged as low risk in 18 of 31 applicable studies, and selective outcome reporting was judged as low risk in all applicable studies. Incomplete outcome data showed mixed judgments, with low risk in 13 studies, high risk in 4 studies, and unclear risk in 14 studies.

### 2.8. Data Synthesis

Studies were included in each qualitative synthesis according to the eligibility criteria and the evidence classification scheme described in Section 2.3 and Section 2.6. The included studies were organized for synthesis by preclinical disease or behavioral model, primary mechanistic target or system, experimental model, and key outcome.

For qualitative synthesis, studies were compared according to disease model, injury induction method, species and animal characteristics, 2-BFI dose and route, treatment timing, evaluation time points, functional outcomes, and tissue, cellular, molecular, inflammatory, or neurovascular outcomes. Particular attention was given to whether functional or behavioral effects were linked to tissue, cellular, molecular, inflammatory, or BBB/NVU-related changes. This approach was used to identify convergent evidence, model-dependent differences, and limitations that precluded quantitative pooling.

Quantitative synthesis was considered, particularly for outcomes reported across multiple ischemic stroke studies, such as infarct volume and neurological scores. However, pooling was not feasible because even these studies differed substantially in stroke model, treatment timing, route and dose of 2-BFI, use of rt-PA or comorbid diabetic conditions, outcome time point, outcome definition, and statistical reporting.

Therefore, no summary effect measure was calculated, and effect estimates were not pooled. Reported outcomes were summarized qualitatively according to the direction and type of 2-BFI-associated effects. The findings were further organized according to mechanisms of 2-BFI-associated neuroprotection, preclinical disease and behavioral models, and cellular and molecular findings.

No data conversion or imputation was performed for synthesis. Formal analyses to explore statistical heterogeneity, such as subgroup analysis or meta-regression, were not performed because the review did not include quantitative meta-analysis. Sensitivity analyses were not conducted because no pooled quantitative synthesis was performed. Formal reporting-bias assessment across studies and certainty-of-evidence assessment were not performed because no quantitative meta-analysis was performed.

## 3. Mechanisms of Neuroprotection by 2-BFI

The mechanistic evidence for 2-BFI spans receptor signaling, oxidative and mitochondrial injury, inflammatory responses, regulated cell death, autophagy, and BBB/NVU integrity [7,8,9,10,11,12,13,14,15,16,17,18,19,20,21,25,26,27,28,39,40,41,42,43,44]. These mechanisms should not be interpreted as isolated pathways. Instead, they represent interconnected secondary injury processes that arise after the initial insult, such as ischemia, mechanical trauma, autoimmune inflammation, amyloid-related toxicity, excitotoxic seizure induction, or cellular stress. Within these secondary injury processes, excitotoxic Ca^2+^ signaling, mitochondrial dysfunction, oxidative stress, immune activation, cell-death pathways, and neurovascular destabilization can amplify tissue damage and worsen neurological or behavioral dysfunction after CNS injury [7,8,9,10,11,12,13,14,15,16,17,18,19,20,21,25,26,27,28,39,40,41,42,43,44]. This section therefore summarizes individual mechanisms and then integrates them into a disease-relevant interpretation of 2-BFI-associated protection.

### 3.1. Pharmacological Identity: I_2_-Site Selectivity and Functional Pharmacology

2-BFI is a high-affinity and selective ligand for imidazoline I_2_ binding sites. Radioligand-binding studies revealed that [^3^H]2-BFI bound brain I_2_ sites with high affinity in a saturable, rapid, and reversible manner, whereas I_1_-site and α_2_-adrenoceptor ligands showed substantially lower affinity for these sites [45]. These binding characteristics established 2-BFI as a reference pharmacological ligand for investigating I_2_-site-associated CNS functions [5,6].

Importantly, the relevance of 2-BFI extends beyond the binding-site characterization. An early focal cerebral ischemia study revealed that 2-BFI treatment reduced infarct volume, neurological deficits, and apoptotic cell death [42], providing evidence that the pharmacological actions of 2-BFI can produce neuroprotective effects in vivo. Electrophysiological studies have subsequently demonstrated rapid and reversible inhibition of N-methyl-D-aspartate receptor (NMDAR)-mediated currents under excitotoxic conditions [43]. The convergence of selective I_2_-site binding, in vivo CNS protection, and a defined functional effect on excitotoxic signaling provides the principal rationale for examining 2-BFI as a neuroprotective I_2_-site ligand.

Functional evidence for I_2_-site involvement has additionally been obtained from antagonist-based pharmacological experiments. Across diverse behavioral and biochemical studies, 2-BFI-associated effects were attenuated or reversed by I_2_-site ligands used as pharmacological antagonists, including idazoxan and BU224. In contrast, blockade of the I_1_ imidazoline receptors with efaroxan or α_2_-adrenoceptors with yohimbine did not consistently suppress these effects [31,32,33,41]. The antagonist findings therefore support I_2_-site involvement more strongly than I_1_ imidazoline or α_2_-adrenergic mechanisms. However, I_2_-site selectivity does not exclude additional pharmacological actions of 2-BFI. For example, MAO-related effects of 2-BFI have been reported in a dopaminergic lesion model [22]. Therefore, I_2_-site binding provides the main pharmacological rationale for this review, but potential non-I_2_ actions and the limitations of antagonist-based inference should be considered when interpreting mechanistic findings. In particular, reversal by idazoxan or BU224 supports I_2_-site involvement but should be interpreted as pharmacological evidence rather than definitive proof of exclusive I_2_-site mediation, because possible non-I_2_ or off-target actions cannot be fully excluded in antagonist-based experiments.

### 3.2. Modulation of NMDA Receptor-Mediated Excitotoxicity

Excessive activation of NMDARs and the subsequent intracellular Ca^2+^ overload are major upstream triggers of excitotoxic neuronal death. However, broad NMDAR blockade has shown limited clinical translation, partly because interference with physiological glutamatergic signaling may produce undesirable CNS effects. Therefore, compounds that preferentially attenuate pathological NMDAR-related signaling while sparing physiological glutamatergic activity may be more relevant in preclinical CNS injury models.

Disease-model evidence provides the primary basis for linking 2-BFI to NMDAR-related neuroprotection. In a rat transient middle cerebral artery occlusion (tMCAO) model combined with NMDAR patch-clamp analysis, Xu et al. [11] revealed that 2-BFI reduced ischemic neuronal injury and preferentially inhibited currents mediated by NMDAR subunit 2B (NR2B)-containing receptors, with limited effects on NMDAR subunit 2A (NR2A)-containing receptors. This pattern supports the preferential attenuation of injury-associated NMDAR signaling during cerebral ischemia.

Additional in vivo evidence extends the NMDAR-related actions beyond ischemic neuronal injury. In experimental autoimmune encephalomyelitis (EAE), Xia et al. [21] showed that 2-BFI reduced neurological deficits and blood–brain barrier (BBB) permeability, preserved occludin expression, and inhibited NMDAR subunit 1 (NR1)/extracellular signal-regulated kinase (ERK) signaling. The association between reduced NR1/ERK signaling and barrier preservation extends NMDAR-related findings to inflammatory neurovascular injuries. In pentylenetetrazol (PTZ)-induced chronic epileptic rats, 2-BFI reduced seizure severity and hippocampal neuronal damage, together with decreased hippocampal caspase-3, NR2A, and NR2B expression [27]. These changes link the modulation of NMDAR expression to the attenuation of excitotoxicity and apoptosis-related injuries in chronic epilepsy.

Cellular and electrophysiological studies have provided mechanistic support for the findings in these disease models. Jiang et al. [44] demonstrated that 2-BFI reduces NMDAR-mediated intracellular Ca^2+^ influx and downstream calpain activation in cultured cortical neurons. Han et al. [43] further showed that 2-BFI rapidly and reversibly inhibited NMDA-evoked currents while sparing α-amino-3-hydroxy-5-methyl-4-isoxazolepropionic acid (AMPA)-evoked responses. This inhibition was noncompetitive and exhibited faster onset and offset kinetics than memantine, indicating that 2-BFI may limit excitotoxic NMDAR activity without prolonged receptor inhibition. However, that study did not directly evaluate whether these kinetic differences translate into improved tolerability or fewer psychotomimetic-like adverse effects compared with memantine. Combined evidence supports the preferential attenuation of pathological NMDAR signaling through inhibition of NR2B-containing receptor currents and reduction in downstream Ca^2+^/calpain activation, with relative preservation of NR2A-containing NMDAR signaling and AMPA-mediated responses. Nevertheless, the available evidence does not establish whether preferential inhibition of NR2B-containing NMDAR currents reflects direct binding of 2-BFI to NR2B-containing receptors or indirect modulation through I_2_-site-associated signaling.

### 3.3. Attenuation of Oxidative Stress and Mitochondrial Injury

Oxidative injury is a major contributor to secondary damage following CNS injury in neurodegeneration-related models. Excessive reactive oxygen species (ROS) accumulation beyond endogenous antioxidant defenses can disrupt mitochondrial activity, induce lipid peroxidation, and intensify apoptosis-related injury pathways. Because imidazoline I_2_ binding sites have been associated with mitochondrial compartments, 2-BFI has been investigated as a modulator of redox homeostasis and mitochondrial integrity.

In a rat spinal cord injury (SCI) model, Lin et al. [17] revealed that 2-BFI improves locomotor recovery and reduces neuronal degeneration in a rat spinal cord injury model. 2-BFI also increased nuclear factor erythroid 2-related factor 2 (Nrf2) and heme oxygenase-1 (HO-1) expression, and enhanced superoxide dismutase (SOD) and glutathione peroxidase (GPx) activities, supporting the activation of endogenous antioxidant defenses after SCI.

A complementary oxidative mechanism has also been identified in diabetic cerebral ischemia–reperfusion injury. For example, Xi et al. [12] showed that 2-BFI reduces NADPH oxidase 2 (NOX2) expression and microglial ROS accumulation. These findings link the antioxidant effects of 2-BFI to the suppression of microglia-derived oxidant production under diabetes ischemic conditions.

Oxidative injury is also attenuated in Alzheimer’s disease (AD)-related pathology. In an amyloid-β (Aβ)-infused AD rat model, Tian et al. [26] revealed that 2-BFI improved learning and memory performance, while reducing hippocampal ROS and malondialdehyde (MDA). These changes were accompanied by increased SOD and GPx activity, linking cognitive improvement to reduced oxidative injury and enhanced antioxidant capacity.

Cellular studies further supported the role of 2-BFI in mitochondrial protection. In primary cultured astrocytes exposed to oxygen–glucose deprivation (OGD), Tian et al. [25] revealed that 2-BFI preserved astrocyte viability, reduced lipid peroxidation, and stabilized the mitochondrial membrane potential, indicating the attenuation of oxidative and mitochondrial dysfunction under ischemia-like metabolic stress.

In fluoride-exposed SH-SY5Y cells and rat primary cortical neurons, Chen et al. [39] showed that 2-BFI maintained the structural stability of the endoplasmic reticulum (ER)–mitochondrial contact sites, also referred to as mitochondria-associated membranes (MAMs). This effect was accompanied by reduced abnormal Ca^2+^ transfer from the ER to the mitochondria, decreased mitochondrial ROS generation, and inhibition of NOD-like receptor family pyrin domain-containing 3 (NLRP3) inflammasome activation. These findings connect the preservation of ER–mitochondria communication with reduced oxidative, inflammatory, and apoptosis-related injury.

Protection against direct oxidative stress has also been observed in hydrogen peroxide (H_2_O_2_)-exposed rat C6 astrocytes, in which 2-BFI attenuated oxidative cytotoxicity and preserved cell viability [40].

Overall, these studies suggest that 2-BFI limits oxidative and mitochondrial damage through coordinated effects on antioxidant defense, microglial and mitochondrial ROS generation, mitochondrial function, and ER–mitochondrial communication. The available studies do not establish whether MAM stabilization represents a direct action of 2-BFI on ER–mitochondria contact sites or a secondary consequence of reduced oxidative and mitochondrial stress. Similarly, increased Nrf2/HO-1 signaling and antioxidant enzyme activities should be interpreted as 2-BFI-associated redox responses rather than evidence that 2-BFI directly activates Nrf2.

### 3.4. Suppression of Neuroinflammation and Modulation of Immune Responses

Neuroinflammation contributes to secondary injury following ischemic stroke, traumatic injury, autoimmune demyelination, and neurodegenerative pathologies. Mechanistic evidence has linked 2-BFI to the modulation of microglial activation, inflammatory signaling, adaptive T-cell balance, inflammasome activation, and inflammatory cell infiltration in CNS models.

In ischemic stroke models, 2-BFI was shown to modulate both inflammatory signaling in injured brain tissue and adaptive immune responses. In diabetic cerebral ischemia–reperfusion injury, improvement in neurological and tissue outcomes are accompanied by reduced microglial activation and proinflammatory cytokine production [12]. These findings identified microglia-associated inflammation as an important component of 2-BFI-mediated protection against diabetic ischemic conditions.

In a rat distal MCAO model, Cheng et al. [13] showed that 2-BFI decreased the phosphorylation of mechanistic target of rapamycin (mTOR) and 70-kDa ribosomal protein S6 kinase (p70S6K), increased interleukin-10 (IL-10) and transforming growth factor-β (TGF-β), and reduced interferon-γ (IFN-γ). This pattern links the 2-BFI-mediated ischemic protection to the suppression of mTOR-related inflammatory signaling and a shift toward anti-inflammatory cytokine responses.

Adaptive immune modulation following MCAO/reperfusion injury has been demonstrated previously. 2-BFI reduces T helper 17 (Th17) cells and interleukin-17A (IL-17A) while increasing regulatory T (Treg) cells and IL-10 [14]. Reciprocal changes in Th17- and Treg-associated responses support the restoration of post-ischemic adaptive immune balance.

Innate immune and inflammasome-related mechanisms are particularly evident following TBI. Post-injury 2-BFI treatment reduced microglial activation and neutrophil infiltration and suppressed interleukin-1β (IL-1β) secretion and NLRP3 inflammasome activation [18]. These findings support the attenuation of microglia- and neutrophil-associated inflammatory injury through the suppression of NLRP3–IL-1β signaling after acute trauma.

In experimental autoimmune encephalomyelitis (EAE), 2-BFI reduced inflammatory cell infiltration and inflammatory pathology in the spinal cord. Long-term treatment additionally decreased tumor necrosis factor-α (TNF-α), IFN-γ, and IL-17A levels together with reduced disease severity [19,20]. These findings extend the immunomodulatory actions of 2-BFI in autoimmune neuroinflammation involving infiltrating immune cells and proinflammatory T-cell-associated cytokines.

Inflammatory and neurotrophic changes were also observed in amyloid-β1–42 (Aβ1–42)-related models. For example, 2-BFI reduced hippocampal TNF-α and interleukin-6 (IL-6) in affective and cognitive impairment models, while brain-derived neurotrophic factor (BDNF) was restored in the cognitive impairment model [24,28]. These changes indicate coordinated modulation of inflammatory and neurotrophic signaling in amyloid-related hippocampal dysfunction.

These studies support the immunomodulatory actions of 2-BFI on innate, adaptive, and tissue inflammatory responses. The evidence converges on the suppression of microglial and NLRP3–IL-1β signaling, modulation of mTOR-related cytokine responses, restoration of Th17/Treg balance, and reduction in inflammatory cell infiltration, together with inflammatory and neurotrophic regulation in amyloid-related models. However, the available evidence does not determine whether Th17/Treg modulation reflects direct effects on T-cell differentiation or indirect changes in antigen presentation, cytokine signaling, or CNS inflammatory injury. Likewise, reduced microglial activation markers should not be interpreted as global microglial suppression, because preservation of beneficial microglial functions such as phagocytosis or debris clearance was not directly tested.

### 3.5. Regulation of Cell Death and Cytoprotective Autophagy

2-BFI-associated cytoprotection involves the attenuation of apoptosis and necroptosis, together with enhancement of lysosome-dependent autophagy. In models of transient cerebral ischemia, Han et al. [42] provided early evidence that 2-BFI reduced apoptotic cell death in the ischemic penumbra, as shown by fewer terminal deoxynucleotidyl transferase dUTP nick-end labeling (TUNEL)-positive neurons. Subsequent molecular analysis further showed that 2-BFI increased B-cell lymphoma 2 (Bcl-2) expression and reduced Bcl-2-associated X protein (Bax) and cleaved caspase-3 [7]. Delayed administration within 5 h of reperfusion retained these anti-apoptotic effects [8]. In an embolic stroke model involving delayed thrombolysis, combined treatment with 2-BFI and a recombinant tissue plasminogen activator (rt-PA) reduced apoptosis-related injury, decreased Bax expression, and increased Bcl-2 expression [15].

Anti-apoptotic effects have also been observed in non-ischemic CNS injury and disease models. For example, in a rat model of SCI, Lin et al. [17] showed that 2-BFI reduced TUNEL-positive neuronal death, increased Bcl-2 levels, and decreased Bax and cleaved caspase-3 levels, supporting the suppression of the mitochondrial apoptosis pathway after SCI. In an Aβ-infused AD rat model, Tian et al. [26] showed that 2-BFI reduced hippocampal apoptosis-related injury. In PTZ-induced chronic epileptic rats, reduced caspase-3 immunoreactivity in the cornu ammonis 1 (CA1), cornu ammonis 3 (CA3), and dentate gyrus regions was accompanied by attenuation of hippocampal neuronal damage [27].

Additional in vivo pharmacological evidence revealed that chronic exposure to I_2_-site ligands, including 2-BFI, reduced the expression of pro-apoptotic proteins in the rat cerebral cortex [41].

Cellular studies have extended the apoptosis-related findings to both astrocytic and neuronal systems. In OGD-exposed primary cortical astrocytes, 2-BFI stabilized the mitochondrial membrane potential, reduced the number of apoptotic cells, and decreased caspase-3 expression [25]. In fluoride-exposed SH-SY5Y cells and primary cortical neurons, 2-BFI preserved cell viability and reduced apoptosis under toxic cellular stress [39].

Evidence from a traumatic brain injury model further suggests that 2-BFI may modulate necroptosis-related neuronal damage. Ni et al. [18] reported that 2-BFI suppressed the receptor-interacting protein kinase 1 (RIPK1)/receptor-interacting protein kinase 3 (RIPK3)/mixed lineage kinase domain-like protein (MLKL) signaling axis, indicating that regulated necrotic cell death may represent another component of 2-BFI-associated protection after acute trauma.

In addition to suppressing cell death, 2-BFI promotes lysosome-dependent autophagy in oxidatively stressed astrocytes. In H_2_O_2_-exposed rat C6 astrocytes, Choi et al. [40] revealed that 2-BFI preserved lysosomal membrane stability and increased the lysosomal localization of mature cathepsin D. 2-BFI also increased microtubule-associated protein 1 light chain 3-II (LC3-II) and lysosome-associated membrane protein 2 (LAMP-2), reduced sequestosome 1 (SQSTM1/p62) accumulation, and enhanced autophagic flux. The lysosomal inhibitor bafilomycin A1 attenuates these cytoprotective and autophagy-related effects, providing functional evidence that lysosome-dependent autophagy and autolysosomal degradation contribute to astrocyte protection.

Overall, available evidence supports the attenuation of apoptosis and necroptosis in animal and cellular models, together with the enhancement of lysosome-dependent autophagy in oxidatively stressed astrocytes. These findings identify regulated cell death and autophagic clearance as important mechanisms underlying 2-BFI-associated cytoprotection. The available studies do not establish whether autophagy enhancement and necroptosis inhibition are mechanistically linked. These effects were observed in different experimental settings and should therefore be interpreted as distinct cytoprotective processes unless a direct interaction between these pathways is demonstrated. Likewise, reduced RIPK1/RIPK3/MLKL phosphorylation may reflect downstream attenuation of oxidative stress, inflammatory signaling, or tissue injury rather than direct inhibition of the necroptosis machinery.

### 3.6. Neurovascular Protection and Combination Therapy with rt-PA

The protective effects of 2-BFI extend from the parenchymal cells to the NVU, in which preservation of neuronal–microvascular interactions and BBB integrity may limit edema and secondary tissue injury. Han et al. [10] showed that 2-BFI preserved neuronal–microvascular coupling during ischemia, suggesting that its neuroprotective effects involve the coordinated preservation of neuronal and microvascular components.

Mechanistic evidence for BBB stabilization was obtained using a rat model of focal cerebral ischemia. 2-BFI preserves occludin, zonula occludens-1 (ZO-1), and collagen IV, while reducing matrix metalloproteinase-9 (MMP-9) expression [9]. These changes support the protection of tight junctions and basement membrane components by attenuating extracellular matrix degradation. Comparable tight junction protection was observed following TBI. Post-injury 2-BFI treatment preserved ZO-1, occludin, and claudin-5 expression in the pericontusional cortex [18], indicating that the barrier-stabilizing effects of 2-BFI were not restricted to cerebral ischemia. In EAE, the preservation of occludin occurs together with the inhibition of NR1/ERK signaling [21], linking receptor-associated signaling to BBB integrity during autoimmune neuroinflammation. Across these models, BBB/NVU findings connect receptor-associated signaling, tight junction preservation, extracellular matrix protection, and inflammatory or traumatic secondary injury responses. However, the included studies do not establish whether tight junction and basement membrane preservation reflects primary endothelial/NVU actions of 2-BFI or secondary protection resulting from reduced inflammatory, oxidative, MMP-related, or tissue injury processes. AQP4-related findings should also be interpreted cautiously, because the included studies did not determine whether increased AQP4 expression reflected beneficial water-clearance responses or potentially maladaptive water movement depending on injury phase, localization, and disease context. The neurovascular effects of 2-BFI are particularly relevant in delayed thrombolysis. In an embolic MCAO model, 2-BFI was administered intravenously at 0.5 h after ischemia, followed by rt-PA at 6 h, to test whether early post-ischemic 2-BFI could extend the preclinical therapeutic window for rt-PA beyond the conventional 4.5-h time frame. This combination reduced infarct volume, neurological deficits, and apoptosis-related injury compared with delayed rt-PA alone. Mechanistically, 2-BFI plus 6-h rt-PA decreased TUNEL-positive cells in the ischemic penumbra, downregulated Bax, and upregulated Bcl-2, indicating attenuation of mitochondrial apoptosis-related injury [15]. These findings provided preclinical evidence that 2-BFI can improve the efficacy of delayed rt-PA and support extension of the rt-PA treatment window to 6 h in an embolic stroke model.

The same embolic MCAO and delayed rt-PA paradigm was subsequently used to define the neurovascular basis of this treatment-window effect [16].

This combination reduced BBB permeability and brain edema, preserved occludin and ZO-1, reduced intercellular adhesion molecule-1 (ICAM-1), matrix metalloproteinase-2 (MMP-2), and MMP-9, while increasing aquaporin-4 (AQP4) expression [16]. These coordinated changes support the attenuation of adhesion- and proteolysis-related barrier disruption, together with the modulation of AQP4-associated water transport. BBB protection was not maintained when rt-PA administration was delayed to 8 h, suggesting that the window-extending effect of 2-BFI remained time-dependent.

The preservation of neuronal–microvascular coupling, tight junction and basement membrane integrity, and suppression of MMP- and ICAM-1-associated barrier injury represent the principal neurovascular mechanisms linked to 2-BFI. The two embolic MCAO studies [15,16] provide complementary evidence that early post-ischemic 2-BFI can extend the preclinical rt-PA treatment window to 6 h. In this paradigm, the window-extending effect was associated with reduced infarct volume, neurological deficits, apoptosis-related parenchymal injury, BBB leakage, edema, tight junction disruption, and MMP- or ICAM-1-associated vascular injury. Together, these findings suggest that 2-BFI may enhance delayed rt-PA therapy by attenuating both apoptotic tissue injury and thrombolysis-associated BBB/NVU damage. The lack of sustained BBB protection at the 8-h rt-PA time point further indicates that this effect is time-dependent and should be interpreted as a defined preclinical window extension rather than an unrestricted expansion of thrombolytic protection.

### 3.7. Integrated Mechanistic Interpretation of 2-BFI-Associated Protection

Across the included studies, 2-BFI-associated protection can be interpreted as modulation of interconnected secondary injury processes after CNS insult. In ischemic and excitotoxic models, attenuation of NMDAR/Ca^2+^ signaling, particularly through preferential inhibition of NR2B-containing NMDAR currents and reduction in Ca^2+^/calpain activation, appears to reduce upstream excitotoxic stress. This mechanism provides a plausible link between early receptor-level modulation and downstream reductions in neuronal injury, apoptosis-related markers, and functional deficits [11,21,27,43,44].

Oxidative and mitochondrial mechanisms provide a second convergent point. In SCI, diabetic ischemia/reperfusion, Aβ-related models, astrocytic OGD, and toxic cellular stress models, 2-BFI enhanced antioxidant defenses, reduced lipid peroxidation or ROS generation, stabilized mitochondrial function, and preserved ER–mitochondria communication. These changes can be connected to inflammatory regulation because mitochondrial and microglial ROS are closely associated with NLRP3 inflammasome activation, cytokine production, and apoptosis-related signaling. Therefore, the antioxidant and mitochondrial effects of 2-BFI may contribute not only to cellular survival, but also to attenuation of inflammatory amplification [12,17,25,26,39,40].

Inflammatory and immune-related findings further connect molecular injury pathways to tissue and functional outcomes. In ischemic stroke, TBI, EAE, and Aβ-related models, 2-BFI reduced microglial activation, neutrophil or inflammatory cell infiltration, proinflammatory cytokine production, NLRP3–IL-1β signaling, and Th17/Treg imbalance. These effects may reflect direct modulation of inflammatory signaling, secondary attenuation of inflammation resulting from reduced tissue injury, or both. The current evidence does not definitively distinguish primary immune actions from indirect anti-inflammatory effects resulting from reduced excitotoxic, oxidative, or vascular damage [12,13,14,18,19,20,21,24,28].

Regulated cell-death pathways represent a shared downstream component of these injury processes. Across ischemic stroke, SCI, AD-related pathology, epilepsy, astrocytic OGD, and toxic cellular stress models, 2-BFI reduced apoptosis-related markers, including TUNEL positivity, Bax/Bcl-2 imbalance, and caspase-3 activation. In TBI, additional evidence implicated suppression of necroptosis-related RIPK1/RIPK3/MLKL signaling. In oxidatively stressed astrocytes, enhancement of lysosome-dependent autophagy suggests that 2-BFI may also support cytoprotective clearance mechanisms. These findings place reduced apoptosis, necroptosis, and impaired autophagic clearance downstream of excitotoxic, oxidative, and inflammatory injury modulation [7,8,15,17,18,25,26,27,39,40,42].

Finally, BBB/NVU protection links molecular and cellular mechanisms to clinically relevant tissue outcomes, including edema, vascular leakage, and thrombolysis-associated injury. Preservation of tight junction proteins, basement membrane components, neuronal–microvascular coupling, and reduced MMP- or ICAM-1-associated barrier injury indicate that 2-BFI-associated protection extends to the neurovascular interface. Overall, the mechanistic evidence supports an integrated interpretation in which 2-BFI attenuates excitotoxic, oxidative, inflammatory, cell-death, and neurovascular injury processes that converge on reduced tissue damage and improved neurological or behavioral outcomes [9,10,16,18,21].

## 4. Disease-Specific Effects of 2-BFI

Preclinical studies on 2-BFI have been conducted in models of ischemic and traumatic CNS injury, autoimmune neuroinflammation, and neurodegeneration-associated pathology, as well as in behavioral and pharmacological models. This section summarizes the disease- and injury-specific functional, behavioral, histopathological, treatment-window, and combination therapy outcomes.

### 4.1. Ischemic Stroke: Acute Protection, Delayed Treatment, and Neurovascular Outcomes

Ischemic stroke is the most extensively studied preclinical disease model for 2-BFI. Across transient focal ischemia, embolic stroke, and diabetic ischemia/reperfusion models, 2-BFI reduced infarct volume and neurological dysfunction. These studies also examined the effects of delayed treatment, neurovascular protection, diabetic comorbidities, and combination therapy with rt-PA [7,8,9,10,11,12,13,14,15,16,42].

Early MCAO studies established the acute in vivo protective effects of 2-BFI, with post-ischemic 2-BFI administration reducing infarct volume and improving neurological outcomes [7,42]. These findings demonstrated that 2-BFI can provide measurable tissue and functional protection after focal cerebral ischemia.

The treatment-window sensitivity was subsequently examined in a transient ischemia/reperfusion model. The administration of 2-BFI during the early post-reperfusion period improves neurological outcomes and reduces ischemic tissue injury, whereas later administration shows diminished efficacy [8]. The accompanying reduction in apoptosis-related injury further linked these functional and tissue benefits to the preservation of ischemic brain tissue.

Stroke-related protection was also later extended to the neurovascular compartments. 2-BFI preserves neuronal–microvascular coupling and cerebral microvascular integrity after focal ischemia [10]. A subsequent study demonstrated reduced BBB leakage, brain edema, and infarct volume, together with the preservation of junctional and basement membrane-associated proteins [9]. Thus, the benefits of 2-BFI in focal ischemia include limiting parenchymal injury and stabilizing the neurovascular interface. Although 2-BFI was administered after experimental ischemia in several stroke studies, these time windows were defined relative to controlled ischemia onset or reperfusion in animal models and should not be directly equated with clinical symptom onset.

Embolic MCAO studies have evaluated 2-BFI under delayed thrombolytic conditions. Administration of 2-BFI 0.5 h after ischemia in combination with rt-PA at 6 h reduced infarct volume, neurological deficits, and apoptosis-related injuries [15]. A subsequent study focused on BBB outcomes, finding reduced BBB permeability and brain edema, together with the preservation of neurovascular integrity, at the 6 h rt-PA time point [16]. These benefits were most evident when rt-PA administration was delayed by 6 h, whereas BBB protection was not maintained when rt-PA administration was delayed by 8 h. This finding indicated that the adjunctive neurovascular benefit of 2-BFI remained time-dependent. Thus, the rt-PA combination evidence supports a preclinical rationale for time-dependent adjunctive thrombolysis studies but does not establish an unrestricted extension of the clinical treatment window. No included study evaluated 2-BFI in combination with mechanical thrombectomy or thrombectomy-related reperfusion injury.

Additional stroke studies have examined diabetic comorbidities and inflammatory, immune, or receptor-related mechanisms. In diabetic ischemia/reperfusion, 2-BFI improved neurological scores and reduced infarct volume, brain edema, neuronal apoptosis, BBB injury, and microglial activation [12]. In distal MCAO and MCAO/reperfusion models, reductions in infarct volume and neurological deficits were accompanied by modulation of mTOR-related cytokine signaling and Th17/Treg balance, respectively [13,14]. A more recent tMCAO study demonstrated reduced infarct volume and neuronal injury together with the preferential inhibition of NR2B-containing NMDAR currents [11].

Taken together, the ischemic stroke studies represent the strongest and most internally connected preclinical evidence cluster for 2-BFI [7,8,9,10,11,12,13,14,15,16,42]. However, this evidence should be interpreted according to model type and treatment context rather than as a uniform stroke dataset. Transient MCAO and ischemia/reperfusion studies mainly support acute neuroprotection and treatment-window sensitivity, with neurological improvement accompanied by reduced infarct volume, apoptosis-related injury, and excitotoxic signaling [7,8,11,42]. In contrast, embolic MCAO studies combined with delayed rt-PA provide a more translationally relevant neurovascular setting, linking 2-BFI to reduced BBB disruption, brain edema, and thrombolysis-associated vascular injury [15,16]. Diabetic ischemia/reperfusion models further extend the evidence to a comorbid inflammatory and oxidative environment [12], whereas distal MCAO and immune-focused MCAO/reperfusion studies emphasize inflammatory signaling and immune balance [13,14]. Thus, although several stroke studies used broadly comparable 2-BFI administration strategies and assessed similar neurological or histological outcomes, differences in ischemia induction, treatment timing, comorbidity status, evaluation time points, and endpoint selection limit direct quantitative comparison. These findings support a qualitative interpretation in which 2-BFI-associated functional improvement is linked to convergent reductions in infarct volume or parenchymal injury, apoptosis, inflammatory amplification, and BBB/NVU disruption [7,8,9,10,11,12,13,14,15,16,42].

### 4.2. Traumatic CNS Injury: Traumatic Brain Injury and Spinal Cord Injury

2-BFI has been evaluated in rat models of TBI and SCI, with treatment initiated after the traumatic insult in both studies. Later post-injury therapeutic windows beyond the early post-injury period were not tested in the included TBI or SCI studies.

In the weight-drop TBI model, 2-BFI treatment was initiated 30 min after the injury, and subsequently administered twice daily for three consecutive days. At 72 h, treatment improved the neurological scores and reduced brain edema, BBB permeability, and cortical tissue loss [18]. These findings show that post-injury 2-BFI treatment improved functional, parenchymal, and neurovascular outcomes after acute traumatic brain injury.

In a clip compression SCI model, twice-daily 2-BFI administration improved hindlimb locomotor recovery during the 21-day observation period and reduced histopathological damage, neuronal degeneration, and apoptosis in the injured spinal cord [17]. These results indicate that 2-BFI promotes functional recovery while limiting secondary neuronal and tissue injury after SCI.

Across the two traumatic injury models, post-injury 2-BFI treatment improved neurological or locomotor function while limiting secondary tissue injury. Although SCI studies included locomotor follow-up, evidence for neuroplasticity, remyelination, axonal repair, or long-term recovery mechanisms remains limited.

### 4.3. Neurodegenerative, Seizure-Related, and Autoimmune Models

#### 4.3.1. Alzheimer’s Disease-Related Models

In Aβ-driven AD-related models, 2-BFI improved cognitive and affective outcomes. In an Aβ-infused AD rat model, 2-BFI improved learning and memory performance and reduced hippocampal pathological changes [26]. These benefits occurred in conjunction with the attenuation of oxidative injury in the hippocampus. An additional Aβ1–42-induced cognitive impairment study revealed that daily 2-BFI treatment prevented deficits in spatial learning and memory and normalized hippocampal Aβ1–42, TNF-α, IL-6, and BDNF immunocontent [28]. Evidence from an Aβ1–42-induced AD-associated depression model extended these findings to affective behavior. 2-BFI reduces depression-like behavior without altering general locomotor activity and modulates hippocampal inflammatory and pathology-related markers [24]. Across these AD-related models, independently administered 2-BFI improved learning, memory, and affective behavior, while reducing hippocampal pathological, oxidative, inflammatory, and apoptosis-related changes. No included study tested 2-BFI in chronic transgenic amyloid models such as APP/PS1 mice or in tauopathy/neurofibrillary tangle models.

#### 4.3.2. Huntington’s Disease-Like Model

In a 3-nitropropionic acid (3-NP)-induced Huntington’s disease (HD)-like rat model, 2-BFI was evaluated in combination with agmatine rather than as an independent treatment. The co-administration of 2-BFI with a subeffective dose of agmatine improved depression-like and motivation-related behaviors and restored the balance between γ-aminobutyric acid (GABA) and glutamate [23]. The combined treatment also reduced pro-inflammatory cytokine levels and restored BDNF expression.

Pharmacological blockade with the imidazoline-site antagonist idazoxan attenuated the behavioral and neurochemical effects associated with agmatine treatment, supporting involvement of imidazoline I_2_-sites. Therefore, these findings support 2-BFI as a modulator or potentiator within agmatine-sensitive signaling in the 3-NP model rather than as independent evidence of disease-modifying efficacy in HD-like pathology.

#### 4.3.3. Parkinsonian 6-Hydroxydopamine Lesion Model

In rats with unilateral 6-hydroxydopamine (6-OHDA) lesions, 2-BFI induced ipsilateral rotation when administered alone, and further enhanced L-3,4-dihydroxyphenylalanine (L-DOPA)-induced contraversive rotation [22]. The ipsiversive response was interpreted to reflect increased dopaminergic activity in the intact hemisphere, whereas the potentiation of L-DOPA-induced rotation may also involve MAO-inhibitory properties of 2-BFI. These findings demonstrate modulation of dopaminergic motor output and L-DOPA responsiveness in the lesioned nigrostriatal system. However, because neuronal survival or lesion preservation were not assessed, this study provides pharmacodynamic evidence for dopaminergic modulation rather than direct evidence of neuroprotection.

#### 4.3.4. Chronic Epilepsy and Hippocampal Injury

In PTZ-induced chronic epileptic rats, 2-BFI reduced seizure severity and attenuated neuronal damage in the hippocampal CA1 and CA3 subfields and dentate gyrus [27]. These findings indicate that 2-BFI protects hippocampal areas vulnerable to repeated seizure-related injuries. The reduction in seizure burden was accompanied by preservation of hippocampal neuronal morphology and decreased apoptosis-related injury.

2-BFI also decreased hippocampal caspase-3 immunoreactivity and NR2A and NR2B expression. These findings indicate that 2-BFI attenuates NMDAR-related excitotoxicity and apoptosis-associated injury in PTZ-induced chronic epileptic rats.

#### 4.3.5. Autoimmune Neuroinflammation and Demyelination in EAE

In EAE models, repeated systemic administration of 2-BFI reduced clinical disease severity, including neurological deficits and hind-limb paralysis, and decreased inflammatory infiltration and spinal cord tissue injury [19,20]. As treatment was administered during disease progression, these studies demonstrated sustained functional and histopathological benefits rather than protection limited to a single acute time point.

A separate EAE study extended these findings to include protection of the BBB. 2-BFI improves neurological status, reduces BBB permeability, and preserves occludin expression [21]. Therefore, EAE studies showed that 2-BFI attenuated clinical disease severity while limiting inflammatory, demyelinating, axonal, and neurovascular injuries. The included EAE studies reported reduced demyelination and axonal injury markers but did not directly test remyelination, axonal repair, or regeneration.

### 4.4. Neuropsychiatric, Pain, Addiction-, and Compulsivity-Related Behavioral Outcomes

#### 4.4.1. Depression- and Anxiety-Like Behavior

In naïve mice, acute 2-BFI administration reduced immobility in both the tail suspension test (TST) and forced swim test (FST) [33]. These effects occurred without any overt reduction in general locomotor activity, supporting an antidepressant-like behavioral effect rather than a nonspecific motor suppression. At higher doses, 2-BFI also showed anxiolytic-like effects, indicating a dose-dependent modulation of both depression- and anxiety-related outcomes. These findings are exploratory behavioral pharmacology and have not been validated in chronic stress, learned helplessness, or other disease-relevant depression models.

#### 4.4.2. Pain- and Opioid-Related Outcomes

In neuropathic and inflammatory pain models, 2-BFI reduced mechanical hypersensitivity as assessed by the von Frey test [31]. Pharmacological manipulation of monoaminergic transmission supports serotonergic and noradrenergic involvement in this antinociceptive effect.

Opioid-related studies have examined adaptations associated with chronic morphine exposure. Chronic co-administration of 2-BFI with morphine attenuates the development of morphine antinociceptive tolerance without acutely potentiating morphine-induced antinociception [34]. In morphine-dependent rats, chronic 2-BFI treatment reduces naloxone-induced locus coeruleus hyperactivity and attenuates morphine tolerance [35]. Thus, opioid-related findings support the modulation of neuroadaptive responses produced by repeated morphine exposure, rather than the enhancement of acute opioid analgesia. These findings suggest a potential opioid-tolerance-modifying action without acute analgesic potentiation, but opioid-sparing efficacy and clinical relevance remain untested.

#### 4.4.3. Alcohol-Related Behavioral Outcomes

Three studies examined ethanol consumption, locomotor sensitization, and withdrawal-associated affective behavior [29,32,36]. In a voluntary ethanol-consumption model, a subeffective dose of 2-BFI potentiated agmatine-induced reductions in ethanol intake and preference, with limited effects on water intake [29]. As 2-BFI was used at a sub-effective dose, this study supports its contribution to the effect of agmatine rather than an independent reduction in ethanol consumption.

In an ethanol sensitization model, 2-BFI reduced both the development and expression of ethanol-induced locomotor sensitization in mice [32]. These findings demonstrate the effects on behavioral plasticity during both the establishment and subsequent expression of repeated ethanol responses.

Following chronic ethanol exposure and abrupt withdrawal, the intracerebroventricular administration of 2-BFI at 20 μg/rat reduced withdrawal-induced depression-like behavior in the FST [36]. A sub-effective dose of 2-BFI potentiated the antidepressant-like effects of agmatine. Alcohol-related studies have identified the effects of 2-BFI on consumption-, sensitization-, and withdrawal-associated behavioral outcomes; however, the consumption study evaluated 2-BFI in combination with agmatine. Thus, alcohol-related findings include both interaction with agmatine-sensitive imidazoline signaling and independent effects on ethanol sensitization or withdrawal-related outcomes, but they do not establish efficacy for reducing alcohol consumption in disease-relevant dependence models.

#### 4.4.4. Compulsivity-Related Behavior

In the marble-burying assay, 2-BFI reduced burying behavior at an effective dose and potentiated the effect of a subeffective dose of agmatine [30]. The treatments did not alter basal locomotor activity. These findings demonstrate the modulation of compulsivity-like behavior without any evidence of nonspecific locomotor suppression and support a pharmacological interaction between 2-BFI and agmatine.

Across these behavioral and pharmacological models, 2-BFI produced antidepressant-, anxiolytic-, and antinociceptive effects, and modified chronic opioid-, alcohol-, and compulsivity-related behaviors. Behavioral evidence also shows that 2-BFI can act independently in selected assays and potentiate agmatine-associated responses when used at subeffective doses.

## 5. Cellular and Molecular Targets of 2-BFI

The included studies have together implicated neurons, astrocytes, microglia, immune responses, BBB/NVU structures, and neurotransmitter-related systems in the protective and modulatory effects of 2-BFI. The following subsections summarize the evidence of these cellular, neurovascular, and neurotransmitter-related components.

### 5.1. Neurons: Protection from Excitotoxicity, Regulated Cell Death, and Axonal Injury

Neuronal protection, reflected by reduced injury and preserved cell survival, is one of the most consistent findings of preclinical studies of 2-BFI. Evidence from ischemic stroke, SCI, TBI, EAE, AD, chronic epilepsy, and toxic injury models indicates that 2-BFI reduces neuronal degeneration, excitotoxic injury, apoptosis, necroptosis, and axonal damage [7,8,9,10,11,12,13,15,16,17,18,19,20,26,27,42]. These in vivo findings are further supported by experiments in cultured neuronal systems [39,43,44].

Across transient, distal, diabetic, and embolic cerebral ischemia models, 2-BFI attenuated neuronal injury through convergent histological and cell-death findings [7,8,9,10,11,12,13,15,16,42]. Hematoxylin and eosin (H&E) staining revealed a reduction in ischemia-induced cell shrinkage, nuclear pyknosis, extracellular space enlargement, and other morphological abnormalities in the injured cortex [8,9,10,11,16]. Nissl staining and neuronal nuclear protein (NeuN) immunostaining further demonstrated the preservation of neuronal populations after distal MCAO [13]. NeuN preservation was interpreted as a marker of neuronal population preservation or survival, not as a standalone functional outcome. 2-BFI also reduces TUNEL-positive apoptotic cells in the infarct and peri-infarct regions [7,8,9,11,12,15,42]. In tMCAO, reduced neuronal injury is accompanied by the preferential inhibition of NR2B-containing NMDAR-mediated signaling [11].

Neuronal protection is also evident following traumatic and inflammatory CNS injuries. Following SCI, 2-BFI reduced Fluoro-Jade B-positive neuronal degeneration and TUNEL-positive cell death and increased Nrf2 immunoreactivity in NeuN-positive neurons, providing cell-specific evidence of neuronal preservation together with enhanced antioxidant signaling [17]. In TBI, treatment reduced necrotic cells detected by H&E staining and propidium iodide (PI)-positive membrane-compromised cells in the pericontusional cortex and attenuated necroptosis-associated neuronal injury [18]. In EAE models, reduced β-amyloid precursor protein (β-APP) accumulation and calpain expression indicated attenuation of spinal cord axonal injury [19,20]. Calpain-related changes were interpreted as downstream markers of calcium-dependent excitotoxic or axonal injury rather than evidence that 2-BFI directly inhibits calpain. Lower cytochrome c and apoptosis-inducing factor expression indicates reduced apoptosis-related injury, whereas reduced demyelination supports the preservation of myelin integrity [19,20].

Neurodegeneration- and seizure-related models provide further evidence of hippocampal neuronal protection. In an Aβ-infused rat model, 2-BFI dose-dependently reduced hippocampal neuronal apoptosis [26]. In PTZ-induced chronic epilepsy, 2-BFI preserved neuronal organization and nuclear morphology, and reduced any intercellular space enlargement in the CA1 and CA3 subfields and dentate gyrus. Treatment also reduced caspase-3-positive neuronal cells in these hippocampal regions [27].

Direct neuronal experiments provide complementary cell-specific evidence. In cultured cortical neurons, 2-BFI reduced glutamate- and NMDA-induced nuclear condensation and neuronal death [42]. In NMDA-exposed primary cortical neurons, treatment preserved neuronal viability and reduced excitotoxic nuclear damage and spectrin proteolysis [40]. In fluoride-exposed SH-SY5Y cells and primary cortical neurons, 2-BFI preserved cell viability and reduced apoptosis-related injury [39].

Together, these findings identified neurons and axons as the major sites of 2-BFI-associated CNS protection. In animal and cellular models, 2-BFI preserves neuronal survival and morphology, reduces apoptosis- and necroptosis-associated injuries, and attenuates axonal degeneration.

### 5.2. Astrocytes: Reactivity, Oxidative Stress, and Autophagy–Lysosome Homeostasis

Astrocyte-related evidence includes in vivo changes in astrocyte reactivity and antioxidant signaling, together with direct cell-based evidence of preserved astrocyte survival, mitochondrial function, and lysosome-dependent autophagy [17,25,26,40].

In a rat SCI model, double-immunofluorescence staining revealed that 2-BFI increased Nrf2 immunoreactivity in glial fibrillary acidic protein (GFAP)-positive astrocytes, providing cell-localized evidence that antioxidant signaling is enhanced in astrocytes after injury [17]. In an Aβ-infused rat model, 2-BFI reduced the AD-associated increase in hippocampal GFAP immunoreactivity, indicating attenuation of astrocyte reactivity in vivo [26].

Direct astrocyte experiments have provided further evidence of cellular protection under ischemia-like metabolic stress. In primary cultured cortical astrocytes exposed to OGD, 2-BFI preserved cell viability and mitochondrial membrane potential while reducing lipid peroxidation. Reductions in apoptosis- and inflammation-related changes further indicate protection against oxidative, mitochondrial, and inflammatory injury [25].

In H_2_O_2_-exposed rat C6 astrocytes, 2-BFI preserved cell viability and lysosomal membrane stability and improved autophagic clearance. Attenuation of these effects by bafilomycin A1 supports a functional contribution of lysosome-dependent autophagy to astrocyte protection [40]. Because bafilomycin A1 attenuated the cytoprotective effect in the astrocyte model, lysosome-dependent autophagic flux appears protective in that experimental setting; however, whether this response is broadly adaptive across CNS disease models remains untested.

Taken together, these findings indicate that astrocytes are a relevant cellular component for 2-BFI-associated protection. Across in vivo and cell-based models, 2-BFI reduced astrocyte reactivity, enhanced antioxidant signaling in astrocytes, and preserved astrocyte survival, mitochondrial function, and autophagy–lysosome homeostasis. Although in vivo studies implicate astrocyte reactivity and astrocyte-localized antioxidant signaling, direct evidence identifying astrocytes as a primary in vivo target of 2-BFI remains limited.

### 5.3. Microglia and Immune Responses: Activation, Infiltration, and Adaptive Immune Balance

The immunomodulatory effects of 2-BFI are reflected in changes in microglial activation, inflammatory-cell infiltration, adaptive T-cell populations, and cytokine expression in injured CNS tissue across ischemic, traumatic, autoimmune, and AD-related models [12,13,14,18,19,20,24,28]. The most direct cell-localized evidence involves microglia and infiltrating immune cells, while cortical and hippocampal cytokine changes provide broader evidence of inflammatory regulation in injured or amyloid-exposed tissues.

In diabetic cerebral ischemia/reperfusion, 2-BFI reduced microglial activation and NOX2 expression and decreased microglia-derived ROS and proinflammatory mediators [12]. These findings identify microglia as a cellular component of the protective response to metabolically vulnerable ischemic conditions.

In a rat distal MCAO model, 2-BFI reduced mTOR and p70S6K phosphorylation in the ischemic cortex and shifted cytokine expression toward an anti-inflammatory pattern, as indicated by increased IL-10 and TGF-β and decreased IFN-γ [13].

Following MCAO/reperfusion, 2-BFI decreased the proportion of Th17 cells and increased the proportion of Tregs, with corresponding changes in IL-17A and IL-10 [14]. These cell-population changes support the restoration of the adaptive immune balance after ischemia. The Th17/Treg findings were derived from a single included study and require independent replication in additional models.

Microglial regulation is also evident following acute trauma. After TBI, 2-BFI reduced activation of ionized calcium-binding adaptor molecule 1 (Iba1)-positive microglia, myeloperoxidase (MPO)-positive inflammatory cell accumulation, and IL-1β expression in the pericontusional cortex. Suppression of microglial NLRP3 inflammasome signaling further links these changes to the attenuation of post-traumatic inflammation [18]. Reduced NLRP3 activation may reflect downstream attenuation of mitochondrial ROS, inflammatory amplification, or tissue injury rather than direct inflammasome inhibition.

In EAE models, repeated 2-BFI treatment reduced inflammatory cell infiltration and inflammatory pathology in the spinal cord [19,20]. These findings extend cellular evidence beyond resident microglia to infiltrating immune populations involved in autoimmune neuroinflammation.

In Aβ1–42-induced affective and cognitive impairment models, 2-BFI reduced hippocampal TNF-α and IL-6 expression, while BDNF was restored in the cognitive impairment model [24,28]. These hippocampal tissue findings indicate inflammatory and neurotrophic regulation, but do not identify any specific immune cell population.

Overall, microglia and infiltrating immune cells are important cellular components of the 2-BFI-associated protection. Cortical and hippocampal cytokine changes and Th17/Treg shifts further indicate a broader regulation of tissue inflammation and adaptive immune responses.

### 5.4. BBB/NVU and Vascular Components: Barrier Integrity and Neurovascular Protection

The BBB/NVU findings identified neuronal–microvascular coupling, tight junction proteins, basement membrane components, barrier-associated proteases and adhesion molecules, and AQP4-related water transport as the principal neurovascular features modulated by 2-BFI [9,10,12,16,18,21].

In focal cerebral ischemia, 2-BFI preserves neuronal–microvascular coupling and cerebral microvascular integrity, demonstrating the protection of the functional NVU [10]. A subsequent MCAO study provided complementary evidence for structural BBB protection. In this model, 2-BFI reduced Evans blue extravasation and brain edema, preserved occludin, ZO-1, and collagen IV expression, and decreased MMP-9 expression [9]. These findings connect the improved barrier function with the preservation of tight junctions and basement membrane components, and reduced extracellular matrix degradation. In diabetic cerebral ischemia/reperfusion, 2-BFI similarly reduced brain edema and BBB injury, extending the neurovascular findings to ischemia in the presence of metabolic comorbidities [12]. In an embolic stroke model involving delayed rt-PA treatment, combined treatment with 2-BFI and rt-PA reduced Evans blue extravasation and brain edema; preserved occludin and ZO-1 expression; and reduced ICAM-1, MMP-2, and MMP-9 expression [16]. Although this increase is consistent with modulation of edema-related water transport, AQP4-related findings should be interpreted cautiously because the included study did not determine whether this change represented beneficial water-clearance regulation or maladaptive water movement [16].

The neurovascular effects of 2-BFI are not restricted to cerebral ischemia. Following TBI, treatment reduces BBB permeability and brain edema and preserves ZO-1, occludin, and claudin-5 in the pericontusional cortex, extending tight junction protection to acute traumatic injury [18]. 2-BFI reduced BBB permeability and preserved occludin expression [21]. Neuronal–microvascular coupling, tight junction proteins, collagen IV, barrier-associated MMPs, ICAM-1, and AQP4-related water transport are the main BBB/NVU-associated components affected by 2-BFI. Across ischemic, traumatic, and autoimmune models, these changes indicate the coordinated preservation of the vascular barrier structure and function. BBB/NVU preservation should be interpreted as one component of a broader protective pattern rather than a mechanism sufficient by itself to explain all functional effects.

### 5.5. Neurotransmitter-Related Systems

Behavioral pharmacology and neurochemical studies link 2-BFI to serotonergic, noradrenergic, dopaminergic, GABA/glutamate, and opioid-related signaling, together with agmatine imidazoline-site interactions [22,23,29,30,31,33,34,35,36]. Neurotransmitter-related findings were derived primarily from behavioral pharmacology, monoamine depletion, receptor antagonism, drug-interaction studies, and limited neurochemical measurements, rather than from direct neurochemical validation across disease models. Involvement of dopaminergic and amino acid neurotransmitters has been observed in various neurodegeneration-related models. In unilateral 6-OHDA-lesioned rats, 2-BFI induced ipsilateral rotation and potentiated L-DOPA-induced contraversive rotation, supporting the modulation of dopaminergic motor output in the lesioned nigrostriatal system [22]. The enhanced L-DOPA response may additionally reflect the MAO-inhibitory properties of 2-BFI and the increased availability of L-DOPA-derived dopamine. In a 3-NP-induced HD-like model, the co-administration of 2-BFI with a sub-effective dose of agmatine improved depression- and motivation-related behaviors and restored the balance between GABA and glutamate [23]. Because 2-BFI was evaluated in combination with agmatine, these findings indicate that 2-BFI potentiates agmatine-associated regulation of amino acid neurotransmission.

Monoaminergic mechanisms have been more directly examined in affective pain models. The antidepressant-like effects of 2-BFI were blocked by idazoxan, methysergide, and haloperidol, implicating I_2_-site, serotonergic, and dopaminergic mechanisms [33]. In neuropathic and inflammatory pain models, 2-BFI-induced antinociception is potentiated by selective monoamine reuptake inhibition and attenuated by monoamine depletion and receptor antagonism. These experiments identified contributions from 5-HT_1_A and 5-HT_2_A receptors and α_1_-adrenoceptors [31]. The same monoaminergic manipulations did not substantially alter the hypothermic or discriminative stimulus effects, indicating that the neurotransmitter systems engaged in the 2-BFI differed according to the behavioral endpoint.

Opioid-related studies have identified the modulation of adaptations produced by repeated morphine exposure. The chronic co-administration of 2-BFI attenuated the development of morphine antinociceptive tolerance without enhancing acute morphine antinociception [34]. In morphine-dependent rats, chronic 2-BFI treatment reduced naloxone-induced locus coeruleus hyperactivity and attenuated neuronal tolerance to morphine [35]. These findings associated 2-BFI treatment with opioid-related adaptations and noradrenergic circuitry involved in dependence and withdrawal.

Agmatine–imidazoline-site interactions were further demonstrated in both alcohol- and compulsivity-related models. Subeffective doses of 2-BFI potentiated agmatine-associated reductions in ethanol intake and preference, antidepressant-like responses during ethanol withdrawal, and reductions in marble-burying behavior [29,30,36]. The sensitivity of these responses to imidazoline receptor antagonism supports the involvement of I_2_-site in agmatine-associated behavioral regulation.

These findings identify the monoaminergic, GABA/glutamate, opioid-related, and agmatine-imidazoline-site pathways as the principal neurotransmitters and neuromodulatory systems linked to 2-BFI. Direct receptor antagonism and neurotransmitter depletion experiments support endpoint-dependent monoaminergic involvement, whereas neurochemical and interaction studies indicate modulation of dopaminergic, GABA/glutamate, opioid-related, and agmatine-sensitive responses. Because monoaminergic, opioid-related, glutamatergic, GABAergic, and agmatine-sensitive systems may have different roles across CNS disorders and behavioral endpoints, these findings should be interpreted as model- and endpoint-specific neuromodulatory pharmacology rather than uniform therapeutic mechanisms.

To summarize the qualitative synthesis and clarify key evidence patterns across model classes, the included studies were organized according to evidence classification, target systems, experimental models, and key functional and mechanistic outcomes (Table 1).

## 6. Discussion

In relation to the established literature on imidazoline receptor biology and I_2_-site pharmacology [4,5,6], this review provides an integrated overview of the preclinical CNS effects of 2-BFI in disease, injury, cellular stress, and behavioral pharmacology models. Rather than converging on a single pathway, available findings link 2-BFI to multiple CNS-relevant processes, including pathological NMDAR signaling, oxidative and mitochondrial injury, neuroinflammation, regulated cell death, BBB/NVU disruption, and neurotransmitter-related behavioral modulation [7,8,9,10,11,12,13,14,15,16,17,18,19,20,21,25,26,27,28,31,32,33,34,35,36,39,40,41,42,43,44]. These effects involve neurons, axons, astrocytes, microglia, infiltrating immune cells, and neurovascular components [7,8,9,10,11,12,13,14,15,16,17,18,19,20,21,25,26,27,28,39,40,41,42,43,44]. Behavioral pharmacology studies have further linked 2-BFI to monoaminergic, GABA/glutamate, opioid-related, and agmatine-sensitive responses [22,23,29,30,31,32,33,34,35,36]. However, the strength and translational relevance of the evidence are not uniform across model classes. Therefore, the findings should be interpreted according to disease model, injury induction method, treatment timing, functional outcome, and the extent to which behavioral or neurological effects are linked to tissue, cellular, molecular, inflammatory, or neurovascular changes.

Among the disease models examined, ischemic stroke provides the most consistent and internally detailed evidence. Across transient focal ischemia, diabetic ischemia/reperfusion, and embolic stroke models, 2-BFI reduces infarct volume or parenchymal injury and neurological dysfunction, and attenuates neuronal, inflammatory, and neurovascular injury [7,8,9,10,11,12,13,14,15,16,42]. Stroke evidence is notable in terms of both the number of models examined and the range of outcomes assessed, including neuronal survival, BBB integrity, brain edema, immune responses, delayed post-reperfusion treatment, and thrombolysis-associated BBB injury. Mechanistic studies have further linked these outcomes to the preferential attenuation of NR2B-containing NMDAR currents, suppression of microglia-derived oxidative and inflammatory responses, modulation of the Th17/Treg balance, and preservation of tight junctions and basement membrane components [9,11,12,13,14]. However, the apparent strength of the stroke evidence should be interpreted cautiously because the studies were conducted in controlled preclinical ischemia models, often with limited reporting of randomization-related procedures and allocation concealment, and publication bias could not be formally assessed. Cellular studies relevant to ischemic and excitotoxic injuries have additionally provided complementary mechanistic support, showing that 2-BFI reduces glutamate- and NMDA-induced neuronal injury and preserves astrocyte viability under OGD-induced metabolic stress [25,44]. Delayed treatment and thrombolysis studies add translational relevance to existing evidence on stroke. The protective effects remained detectable when 2-BFI was administered following reperfusion, although its efficacy declined with increasing delay [8]. In embolic stroke, early 2-BFI administration improved outcomes when rt-PA was delayed to 6 h and reduced the associated BBB and edema-related injuries [15,16]. Although BBB protection was not maintained when rt-PA was delayed to 8 h, the observed benefit under delayed reperfusion conditions supports the potential value of 2-BFI as an adjunctive strategy to reduce neurovascular injury associated with reperfusion therapy. The loss of BBB protection at the 8 h rt-PA time point indicates that the adjunctive thrombolysis evidence is time-dependent and may reflect a relatively narrow preclinical therapeutic window, which could limit clinical utility unless dosing, timing, safety, and reperfusion conditions are more precisely defined. Thus, the stroke evidence is strongest when functional improvement is interpreted together with reductions in infarct volume, apoptosis-related injury, inflammatory amplification, and BBB/NVU disruption, rather than as isolated neurological score improvement.

Evidence from traumatic, autoimmune, and seizure-related CNS injury models extends the 2-BFI-associated protection beyond cerebral ischemia, although there are few available studies. In TBI and SCI, post-injury treatment improved neurological or locomotor function and reduced secondary tissue injury [17,18]. TBI studies are especially informative because inflammatory activation, necroptosis-associated neuronal injury, and BBB disruption were evaluated using the same model [18]. Studies on SCI have linked functional recovery and neuronal preservation to antioxidant and anti-apoptotic responses [17]. In EAE, repeated systemic treatment reduces disease severity, inflammatory infiltration, demyelination, axonal injury, and BBB disruption [19,20,21]. Chronic epilepsy provided additional evidence that 2-BFI can reduce seizure severity and preserve hippocampal neuronal structure in a model of repeated excitotoxic injury [27]. Taken together, these studies suggest that 2-BFI-associated protection extends to secondary injury processes shared by traumatic, autoimmune, and seizure-related CNS disorders. Nevertheless, compared with ischemic stroke, these disease areas are supported by fewer studies and should be viewed as promising but less extensively replicated evidence.

Neurodegeneration-related studies require model-specific interpretations as 2-BFI is used differently across AD-related, HD-like, and Parkinsonian lesion models. In Aβ-based AD-related models, independently administered 2-BFI improved learning, memory, or depression-like behavior and reduced hippocampal pathological, inflammatory, oxidative, and apoptosis-related changes [24,26,28]. These findings support 2-BFI-associated effects on amyloid-related cognitive and affective dysfunctions. In a 3-NP-induced HD-like model, 2-BFI was evaluated in combination with agmatine to potentiate the agmatine-associated behavioral and neurochemical responses [23]. In a unilateral 6-OHDA lesion model, 2-BFI modulated rotational behavior and L-DOPA responsiveness; however, neuronal survival and lesion preservation were not assessed [22]. Thus, AD-related studies support direct 2-BFI-associated effects in amyloid-related models, whereas HD-like and Parkinsonian studies primarily support pharmacological modulation rather than independent disease-modifying efficacy. No included study evaluated 2-BFI in tauopathy models or directly assessed tau pathology or neurofibrillary tangle-related outcomes.

Additional behavioral studies show that 2-BFI influences affective-, pain-, opioid-, alcohol-, and compulsivity-related outcomes, but the strength and interpretation of neurotransmitter-related evidence differ across systems. The most direct behavioral pharmacology evidence supports monoaminergic modulation, because antidepressant- and antinociceptive-like effects were supported by antagonist, monoamine-depletion, and reuptake-manipulation experiments involving serotonergic and noradrenergic mechanisms [31,33]. Opioid-related findings are more specifically linked to neuroadaptive responses after repeated morphine exposure, including antinociceptive tolerance and locus coeruleus hyperactivity, rather than acute potentiation of opioid analgesia [34,35]. Dopaminergic findings in the 6-OHDA lesion model demonstrate modulation of nigrostriatal motor output and L-DOPA responsiveness, but they should be interpreted as pharmacodynamic evidence because neuronal preservation was not assessed [22]. GABA/glutamate-related findings were mainly observed in the 3-NP model with agmatine co-treatment and therefore provide model-specific evidence for excitatory–inhibitory balance rather than a broadly established mechanism of 2-BFI alone [23].

Repeated agmatine-related findings can be integrated as evidence for imidazoline-sensitive modulatory signaling rather than as separate disease-specific effects. Across HD-like, ethanol-consumption, ethanol-withdrawal, and compulsivity-related models, 2-BFI either potentiated subeffective agmatine responses or showed interactions consistent with imidazoline-site involvement [23,29,30,36]. These findings suggest that agmatine-related experiments primarily support a unified interpretation of 2-BFI as an enhancer of agmatine-sensitive imidazoline-related behavioral and neurochemical responses. However, the available agmatine-combination studies do not establish whether 2-BFI acts as an allosteric modulator, direct agonist, or permissive modulator of agmatine-associated I_2_-site signaling. Accordingly, these studies should be distinguished from disease-modifying neuroprotective evidence unless accompanied by tissue, cellular, or pathology-related protection.

Taken together, the available evidence indicates that 2-BFI has broad preclinical CNS relevance across injury, neurodegeneration-related, and behavioral pharmacology models [7,8,9,10,11,12,13,14,15,16,17,18,19,20,21,22,23,24,25,26,27,28,29,30,31,32,33,34,35,36,39,40,41,42,43,44]. The strongest therapeutic-development rationale is currently concentrated in ischemic stroke and neurovascular injury, where functional outcomes are repeatedly linked to tissue protection, apoptosis-related changes, inflammatory modulation, and BBB/NVU stabilization [7,8,9,10,11,12,13,14,15,16,42]. Traumatic CNS injury, autoimmune neuroinflammation, seizure, and AD-related models extend the biological relevance of 2-BFI but remain less replicated or more model-specific [17,18,19,20,21,24,26,27,28]. Behavioral, opioid-, alcohol-, compulsivity-, and agmatine-related studies further support CNS neuromodulatory actions, but they should not be interpreted as equivalent to disease-modifying neuroprotection [22,23,29,30,31,32,33,34,35,36]. This distinction is important for balancing the therapeutic interpretation of 2-BFI and for identifying where future preclinical development should be prioritized.

From a cellular and mechanistic perspective, the available evidence suggests that functional improvement after 2-BFI treatment is most convincing when neurological or behavioral outcomes are accompanied by tissue, cellular, molecular, inflammatory, or neurovascular changes. In ischemic stroke models, improved neurological scores were repeatedly associated with reduced infarct volume, apoptosis-related injury, inflammatory responses, edema, and BBB/NVU disruption [7,8,9,10,11,12,13,14,15,16,42]. In SCI, TBI, AD-related, EAE, and epilepsy models, behavioral or neurological improvement was also accompanied by changes in oxidative stress, inflammatory signaling, neuronal survival, demyelination, seizure-related hippocampal injury, or barrier integrity [17,18,19,20,21,24,26,27,28]. These findings support a functional–mechanistic interpretation in which 2-BFI-associated behavioral or neurological benefits are linked to reductions in secondary tissue injury rather than to isolated behavioral modulation alone.

Cell-type-specific findings indicate that neurons are the most direct readout of 2-BFI-associated protection, particularly in models reporting reduced excitotoxic signaling, apoptosis, infarct volume, neuronal degeneration, or hippocampal injury [7,8,11,15,17,25,27,42,44]. Astrocyte-related evidence extends this interpretation by implicating oxidative stress control, mitochondrial stabilization, lysosome-dependent autophagy, and support of tissue homeostasis under metabolic or oxidative stress [25,39,40]. Microglial and immune-related findings suggest that 2-BFI may attenuate inflammatory amplification through reduced microglial activation, ROS production, NLRP3–IL-1β signaling, cytokine responses, and Th17/Treg imbalance [12,13,14,18,19,20,21,24,28]. Neurovascular findings further indicate that endothelial and BBB/NVU-related responses contribute to edema control, vascular leakage reduction, and preservation of tight junction and basement membrane components [9,10,16,18,21]. Thus, the current evidence supports a multicellular interpretation in which neuronal, astrocytic, immune, and neurovascular responses jointly contribute to 2-BFI-associated functional protection.

The relative importance of these cell types appears to differ by model. In ischemic and excitotoxic injury, neuronal NMDAR/Ca^2+^ signaling and apoptosis-related injury are closely linked to infarct volume and neurological dysfunction [7,8,11,15,25,27,42,44]. In oxidative or metabolic stress models, astrocyte mitochondrial stability and autophagy-related clearance may be especially relevant [25,39,40]. In diabetic ischemia, TBI, EAE, and Aβ-related models, microglial activation, infiltrating immune cells, cytokine production, and adaptive immune balance become more prominent [12,18,19,20,21,24,28]. In embolic stroke with delayed rt-PA, BBB/NVU stabilization is particularly important because the therapeutic effect is linked to reduced vascular leakage, edema, and MMP- or ICAM-1-associated barrier injury [15,16].

At present, however, the included studies do not definitively establish whether immune modulation is a primary pharmacological action of 2-BFI or a secondary consequence of reduced tissue injury. Decreased microglial activation, cytokine production, or immune-cell infiltration may reflect direct modulation of inflammatory signaling, but these changes may also result from smaller lesions, reduced excitotoxic injury, improved mitochondrial stability, or preserved BBB/NVU integrity [12,13,14,18,19,20,21,24,28]. Future studies using cell-specific approaches, conditional models, or isolated cell systems aligned with in vivo outcomes will be needed to define whether neurons, astrocytes, microglia, endothelial cells, or infiltrating immune cells represent primary targets of 2-BFI in each disease setting.

To clarify the relative strength of evidence, mechanistic and outcome linkages, key uncertainties, and translational implications across model classes, Table 2 summarizes the integrated interpretation of the preclinical 2-BFI evidence.

Several limitations may affect the interpretation of this literature. First, the included studies differed substantially in the model type, dosing regimen, treatment timing, route of administration, and outcome selection, which limited direct comparisons and precluded a summary estimate of efficacy. Dose, route, treatment timing, and evaluation time points varied substantially across studies. Although 3 mg/kg was repeatedly used in several ischemic and neurovascular injury studies, other studies used broader systemic dose ranges, intracerebroventricular microgram doses, repeated behavioral dosing paradigms, or in vitro micromolar to millimolar concentrations. Routes included intravenous, intraperitoneal, intracerebroventricular, subcutaneous, and direct cellular or tissue application. Treatment and assessment schedules ranged from pretreatment or immediate post-insult administration to delayed post-reperfusion treatment, repeated disease-course dosing, real-time cellular measurements, 24–72 h acute injury outcomes, 7–28 d disease-course assessments, and acute post-drug behavioral testing. These differences indicate that dose–response relationships, therapeutic index, CNS exposure, exposure–response profiles, and clinically relevant therapeutic windows remain insufficiently defined. This heterogeneity represents a major obstacle for translation because it prevents direct comparison of efficacy across models and makes it difficult to define a reproducible dose, route, treatment window, and outcome set for subsequent preclinical development.

The animal characteristics of the included in vivo studies also limit generalizability. Among studies with reported sex, most used male animals. Female animals were represented primarily in the three EAE studies [19,20,21], and one behavioral study included animals of both sexes [29]. Rats and mice were both represented, and animal characteristics were reported heterogeneously, including age ranges from approximately 8 weeks to 4 months, descriptions such as adult or healthy adult, and body-weight-based reporting without precise age information. This male-predominant and variably reported animal profile, together with the limited use of aged, comorbid, or sex-balanced designs, may reduce the translational generalizability of the current preclinical evidence.

In addition, the strength of cell-specific evidence varied. Neuronal and astrocyte studies have included direct cellular experiments and co-localization analyses, whereas several immune and BBB/NVU findings were derived from injured tissues, barrier permeability, or protein expression measurements that did not identify the responsible cell population. Many disease studies have focused on acute or subacute outcomes, whereas long-term functional recovery has been evaluated less consistently. Behavioral outcomes, such as rotational activity, opioid tolerance, alcohol sensitization, and marble burying, are also assay-defined measures that do not directly correspond to clinical diagnoses. Outcome measures ranged from tissue and molecular injury markers to disease-specific behavioral assays and short-term cellular readouts, further limiting direct cross-model comparison.

Incomplete reporting of key methodological procedures in the included studies was another limitation. The risk of bias was assessed using the SYRCLE tool for eligible in vivo animal studies. However, randomization-related procedures, allocation concealment, random housing, and blinding of caregivers or investigators were not consistently reported. In vitro-only studies were not formally assessed using SYRCLE’s tool, which is designed for animal intervention studies; therefore, the methodological appraisal of cellular studies was limited. Regarding the review process, the protocol was not prospectively registered. The conclusions also reflect literature available up to the final search date of May 19, 2026, and studies published thereafter may further refine the evidence classification or translational interpretation. Formal reporting-bias assessment was not performed because of the qualitative and heterogeneous nature of the included evidence. Certainty-of-evidence grading was not performed because of the substantial heterogeneity in the models, interventions, and outcomes. Human pharmacokinetic, brain-exposure, target-engagement, and safety data for 2-BFI also remain limited, and most studies did not systematically define repeated-dose tolerability or therapeutic index. No eligible study provided a complete pharmacokinetic/pharmacodynamic profile, oral bioavailability, brain penetration, half-life, or exposure–response relationship for 2-BFI in the CNS disease models reviewed. Therefore, the conclusions should be interpreted as a qualitative synthesis of preclinical findings, rather than a quantitative or graded estimate of therapeutic efficacy.

Future studies should prioritize following standardized disease-specific designs with comparable dosing, treatment timing, routes of administration, and functional outcomes. Pharmacokinetic and target engagement analyses are required to connect systemic exposure to CNS I_2_-site engagement. Dose–response relationships, therapeutic windows, brain-exposure profiles, repeated-dose safety, aged and comorbid animal models, and sex-balanced designs should be incorporated before clinical translation is considered. Cell-specific causal experiments are required to clarify whether neuronal, astrocytic, microglial, immune, or neurovascular responses occur directly or secondary to reduced injury. Studies on stroke should further define the therapeutic window and safety of 2-BFI in combination with reperfusion therapy, particularly because the current rt-PA combination evidence supports a defined preclinical window extension rather than unrestricted expansion of thrombolytic protection. Chronic neurodegenerative and behavioral studies should distinguish independent 2-BFI effects from the potentiation of agmatine or other treatments and include disease-relevant structural outcomes when neuroprotection is proposed.

## 7. Conclusions

Overall, this systematic review synthesized preclinical evidence indicating that 2-BFI has broad CNS relevance across disease, injury, cellular stress, and behavioral pharmacology models. The most consistent evidence was found in ischemic stroke and neurovascular injury models, in which 2-BFI reduced neurological deficits and tissue injury, preserved neuronal and neurovascular integrity, and showed protective activity within defined delayed-treatment and rt-PA combination settings. Additional studies provide supportive but less extensively replicated evidence in traumatic injury, EAE, chronic epilepsy, and AD-related models, whereas HD-like, Parkinsonian, and behavioral studies primarily provide pharmacological evidence involving neurotransmitter-related modulation and treatment-interaction responses.

Across the included studies, 2-BFI-associated effects involved pathological NMDAR signaling, oxidative and mitochondrial injury, neuroinflammation, apoptosis and necroptosis, astrocyte autophagy–lysosome function, and BBB/NVU disruption. These findings support the interpretation of 2-BFI as a promising preclinical imidazoline I_2_-site ligand with relevance to multiple CNS injury-related and neuromodulatory processes, rather than as an established therapeutic agent or as a compound acting through a single established pathway. Although the heterogeneity in models, interventions, outcome measures, and methodological reporting requires cautious interpretation, the available evidence supports further disease-focused preclinical evaluation of 2-BFI. Standardized replication, pharmacokinetic, brain-exposure, safety, and target-engagement studies, cell-specific causal experiments, aged and comorbid animal models, sex-balanced designs, and long-term functional assessments are required to define the translational potential of 2-BFI in CNS disorders.

Based on the current strength of evidence, ischemic stroke and neurovascular injury represent the most appropriate priority areas for disease-focused preclinical development of 2-BFI. Future studies in this area should use standardized ischemic and reperfusion models, delayed post-onset treatment designs, predefined BBB/NVU and functional outcomes, dose–response analyses, pharmacokinetic and brain-exposure measurements, and target-engagement assays. Traumatic CNS injury, EAE, chronic epilepsy, and AD-related models should be considered secondary preclinical areas that require independent replication, disease-relevant long-term outcomes, and model-specific validation before similar prioritization. Behavioral, opioid-, alcohol-, compulsivity-, and agmatine-related findings should currently be regarded as exploratory neuromodulatory pharmacology rather than robust evidence for disease-focused therapeutic development. Therefore, clinical translation should proceed only after preclinical PK/PD, brain-exposure, repeated-dose safety, target-engagement, and exposure–response studies establish a reproducible therapeutic window and clinically relevant efficacy profile.

## Figures and Tables

**Table 1:** Study-level evidence map of 2-BFI across preclinical CNS models, target systems, experimental models, and key functional and mechanistic outcomes. Studies are grouped by evidence classification to distinguish the strongest ischemic stroke and neurovascular evidence cluster from supportive traumatic, neuroinflammatory, neurodegeneration-related, behavioral, and cellular mechanistic evidence. Outcomes are summarized to highlight functional or behavioral effects together with associated tissue, cellular, molecular, inflammatory, neurotransmitter-related, or BBB/NVU-related findings. The value of n indicates the number of included studies in each evidence classification.

Evidence Classification	Target/System	Experimental Model	Key Functional and Mechanistic Outcomes	Study /Reference
Ischemic stroke and neurovascular outcomes (n = 13)	Neurons	Rat transient MCAO	Reduced infarct volume, neurological deficits, TUNEL-positive cells, and subcellular injury, with increased Bcl-2 expression.	Han et al. (2010) [7]
		Rat MCAO/reperfusion	Defined an early post-reperfusion therapeutic window, with reduced neurological deficits, infarct volume, and apoptosis-related injury through Bcl-2/Bax modulation.	Zhang et al. (2018) [8]
		Rat tMCAO	Reduced neuronal injury and preferentially inhibited NR2B-containing NMDAR currents, linking protection to NR2B-related excitotoxic signaling.	Xu et al. (2024) [11]
		Rat embolic MCAO	Combined 2-BFI and delayed rt-PA reduced infarct volume, neurological deficits, and apoptosis-related injury through Bax/Bcl-2 modulation.	Guo et al. (2018) [15]
		Rat transient MCAO	Improved neurological scores and reduced infarct volume, cleaved caspase-3, and TUNEL-positive cells.	Han et al. (2009) [42]
		Cultured cortical neurons	Reduced glutamate- and NMDA-induced excitotoxic injury by reversibly inhibiting NMDAR-mediated Ca^2+^ influx and calpain activation.	Jiang et al. (2010) [44]
	BBB/NVU	Rat MCAO/reperfusion	Reduced infarct volume, apoptosis-related injury, edema, and BBB leakage while preserving occludin, ZO-1, and collagen IV and suppressing MMP-9.	Zhang et al. (2018) [9]
		Rat MCAO	Improved neurological deficits and reduced infarct volume while preserving neuronal–microvascular coupling, vWF expression, and cerebral microvascular integrity.	Han et al. (2012) [10]
		Rat embolic MCAO	Reduced infarct volume, BBB permeability, edema, and denatured cell injury while preserving AQP4, occludin, and ZO-1 in the delayed rt-PA setting.	Zhang et al. (2020) [16]
	Adaptive immune balance	Rat MCAO/reperfusion	Reduced infarct volume and neurological deficits while restoring Th17/Treg balance, decreasing IL-17A, and increasing IL-10.	Huang et al. (2020) [14]
	Inflammatory signaling	Rat distal MCAO	Improved neurological deficits and reduced infarct volume, with suppression of mTOR/p70S6 signaling and a shift toward anti-inflammatory cytokine responses.	Cheng et al. (2021) [13]
	Microglia	Diabetic rat MCAO/reperfusion	Reduced neurological injury, infarct volume, edema, apoptosis-related injury, BBB damage, and microglial activation.	Xi et al. (2021) [12]
	Astrocytes	Primary astrocytes OGD	Preserved astrocyte viability by reducing lipid peroxidation, stabilizing mitochondrial function, and attenuating apoptosis-related injury.	Tian et al. (2018) [25]
Acute traumatic CNS injury (n = 2)	Microglia, neurons, and BBB/NVU	Rat focal cortical contusion TBI	Improved neurological outcomes and reduced edema, BBB permeability, tissue loss, inflammatory infiltration, NLRP3 activation, and necroptosis-related signaling.	Ni et al. (2019) [18]
	Spinal cord neurons	Rat clip-compression SCI	Improved locomotor recovery while enhancing Nrf2/HO-1 and antioxidant enzyme activity and reducing neuronal degeneration and apoptosis-related injury.	Lin et al. (2019) [17]
Neuroinflammatory, neurodegeneration-related, and seizure models (n = 9)	Axon/myelin	Mouse MOG_35–55_-induced EAE	Improved neurobehavioral scores and myelin preservation while reducing axonal injury, calpain activation, and metabolic enzyme disruption.	Wang et al. (2011) [20]
	Immune–axonal injury	Mouse MOG_35–55_-induced EAE	Reduced disease severity and hind-limb paralysis, with decreased proinflammatory cytokines, inflammatory cell infiltration, and neuronal injury markers.	Zhu et al. (2015) [19]
	BBB/NVU	Mouse MOG_35–55_-induced EAE	Reduced neurological deficits and BBB permeability while preserving occludin and inhibiting NR1/ERK signaling.	Xia et al. (2021) [21]
	Hippocampal neurons	Rat hippocampal Aβ injection	Improved learning and memory performance while reducing hippocampal oxidative stress, inflammation, and apoptosis-related injury.	Tian et al. (2017) [26]
		Mouse Aβ depression-like phenotype	Reduced depression-like behavior and hippocampal TNF-α/IL-6 expression, with additional potentiation of agmatine-associated behavioral effects.	Kotagale et al. (2020) [24]
		Mouse Aβ memory impairment	Prevented memory deficits and normalized hippocampal Aβ1–42, TNF-α, IL-6, and BDNF immunocontent.	Kotagale et al. (2020) [28]
		Rat PTZ-induced chronic epilepsy	Reduced seizure severity and hippocampal neuronal damage, with decreased caspase-3, NR2A, and NR2B expression.	Tu et al. (2016) [27]
	Neurotransmitter systems	Rat 3-NP HD-like phenotype	Potentiated agmatine-associated behavioral and neurochemical effects, improving affective/motivation-related behaviors and normalizing GABA/glutamate balance, cytokines, and BDNF.	Katariya et al. (2026) [23]
		Rat 6-OHDA lesion	Modulated dopaminergic motor output and L-DOPA responsiveness, without direct assessment of neuronal preservation.	Macinnes & Duty (2004) [22]
Neuropsychiatric, pain, addiction-, and compulsivity-related phenotypes (n = 8)	Neurotransmitter systems	Naïve mouse affective assays	Reduced depression- and anxiety-like behaviors without altering locomotor activity, with pharmacological evidence supporting I_2_-site and monoaminergic involvement.	Tonello et al. (2012) [33]
		Rat pain models	Reduced neuropathic and inflammatory pain behaviors, with monoaminergic involvement supported by depletion, receptor blockade, and reuptake-manipulation experiments.	Siemian et al. (2018) [31]
		Rat ethanol intake	Potentiated agmatine-induced reductions in ethanol self-administration and voluntary ethanol consumption, supporting agmatine-sensitive imidazoline-related behavioral modulation.	Taksande et al. (2019) [29]
		Mouse ethanol sensitization	Reduced development and expression of ethanol-induced locomotor sensitization and potentiated agmatine-associated inhibitory effects, supporting imidazoline-sensitive behavioral modulation.	Taksande et al. (2019) [32]
		Rat ethanol withdrawal	Reduced ethanol withdrawal-induced depression-like behavior and potentiated agmatine-associated antidepressant-like responses.	Chimthanawala et al. (2020) [36]
		Mouse marble-burying	Reduced marble-burying behavior without altering locomotor activity and potentiated subeffective agmatine-associated anticompulsive-like effects.	Dixit et al. (2014) [30]
	Opioid system	Rat morphine tolerance	Attenuated morphine antinociceptive tolerance after chronic I_2_-ligand co-administration without potentiating acute morphine antinociception.	Boronat et al. (1998) [34]
		Rat morphine dependence	Attenuated naloxone-induced locus coeruleus hyperactivity and morphine tolerance after chronic co-administration.	Ruiz-Durántez et al. (2003) [35]
Cellular and organelle-specific mechanisms (n = 4)	Mitochondria/ER	Neuronal fluorosis cells	Preserved neuronal cell viability by stabilizing ER–mitochondria contact sites, reducing mitochondrial Ca^2+^/ROS dysregulation, and inhibiting NLRP3 inflammasome activation.	Chen et al. (2023) [39]
	Cortical neurons	Rat chronic I_2_-ligand treatment	Shifted cortical apoptosis-related protein expression toward an anti-apoptotic balance after chronic selective I_2_-ligand treatment.	Garau et al. (2013) [41]
	Neurons	Rat primary cortical neurons	Transiently inhibited NMDA-evoked currents without affecting AMPA currents and reduced NMDA-mediated Ca^2+^ entry and excitotoxic neuronal injury.	Han et al. (2013) [43]
	Astrocyte autophagy	C6 astrocyte oxidative stress	Protected astrocytes against oxidative injury by enhancing lysosomal stability, LC3-II conversion, and autolysosomal degradation.	Choi et al. (2018) [40]

**Table 2:** Integrated interpretation of 2-BFI evidence across model classes, mechanisms, limitations, and translational relevance. Evidence areas are summarized to highlight relative evidence strength, mechanistic and outcome linkages, key limitations or uncertainties, and translational interpretation and next steps across preclinical CNS models.

Evidence Area	Evidence Strength	Mechanistic and Outcome Linkage	Key Limitations/Uncertainties	Translational Interpretation and Next Steps
Ischemic stroke and neurovascular injury	Strongest and most internally connected evidence	Neurological improvement was linked to reduced infarct injury, apoptosis, excitotoxic signaling, inflammation, edema, BBB leakage, and BBB/NVU disruption.	Controlled preclinical models; incomplete reporting of some bias-related methods; publication bias not formally assessed; rt-PA benefit was time-dependent and not sustained at 8 h.	Priority area for disease-focused preclinical development; requires standardized ischemia/reperfusion models, delayed dosing, reperfusion-combination studies, PK/brain-exposure, target engagement, and long-term outcomes.
Traumatic CNS injury	Supportive but limited	Functional improvement was associated with reduced oxidative stress, apoptosis, necroptosis, NLRP3 signaling, edema, tissue loss, and blood–CNS barrier disruption.	Evidence limited to two injury models; later treatment windows, long-term recovery, neuroplasticity, remyelination, and axonal repair remain insufficiently tested.	Preliminary supportive area; requires independent replication, delayed-treatment studies, long-term functional assessment, and mechanistic validation.
Neuroinflammatory, neurodegeneration-related, and seizure models	Supportive but heterogeneous and model-specific	Behavioral, cognitive, seizure, or motor outcomes were linked to immune modulation, BBB protection, axon/myelin preservation, hippocampal injury reduction, and neurotransmitter-related changes.	AD evidence is limited to amyloid-related models; tauopathy was not tested; EAE studies did not directly assess remyelination or regeneration; some findings reflect treatment interactions.	Biologically relevant, but further disease-focused development should be supported by disease-specific replication, structural pathology outcomes, and separation of independent 2-BFI effects from treatment-interaction effects.
Neuropsychiatric, pain, addiction-, and compulsivity-related phenotypes	Exploratory neuromodulatory pharmacology	Behavioral effects involved monoaminergic, opioid-related, ethanol-related, and agmatine-sensitive imidazoline-related modulation, mostly without tissue-protective endpoints.	Disease-relevant models and direct neurochemical validation are limited; opioid-sparing efficacy, dependence models, and depression models remain insufficiently tested.	Should be interpreted as exploratory neuromodulatory pharmacology rather than robust evidence for disease-focused therapeutic development.
Cellular mechanisms across CNS models	Mechanistic support	Cellular protection was linked to ER–mitochondria stabilization, NMDAR/Ca^2+^ modulation, NLRP3-related signaling, apoptosis-related proteins, and lysosome–autophagy responses.	Cell-based findings require validation in disease-relevant in vivo models; direct versus secondary actions remain unresolved for several pathways.	Supports mechanistic plausibility; requires cell-specific causal experiments, target-engagement assays, and alignment with functional outcomes in vivo.

## Data Availability

No new data were created or analyzed in this study. Data sharing is not applicable to this article.

## Supplementary Materials

The following supporting information can be downloaded at: https://www.mdpi.com/article/10.3390/ph19081155/s1, Table S1: Database-specific search strategies and records retrieved; Table S2A: Category-level summary of excluded reports; Table S2B: Record-level list of excluded reports with primary reasons for exclusion; Table S3A: Study-level SYRCLE risk-of-bias assessment for the included studies; Table S3B: SYRCLE domain-level summary of risk-of-bias judgments.

## Abbreviations

The following abbreviations are used in this manuscript:

2-BFI	2-(2-benzofuranyl)-2-imidazoline
3-NP	3-nitropropionic acid
5-HT	5-hydroxytryptamine
6-OHDA	6-hydroxydopamine
AD	Alzheimer’s disease
AIF	Apoptosis-inducing factor
AMPA	α-amino-3-hydroxy-5-methyl-4-isoxazolepropionic acid
AQP4	Aquaporin-4
Aβ	Amyloid-β
Bax	Bcl-2-associated X protein
BBB	Blood–brain barrier
Bcl-2	B-cell lymphoma 2
BDNF	Brain-derived neurotrophic factor
B-CK	Brain-type creatine kinase
Ca^2+^-ATPase	Calcium ATPase
CNS	Central nervous system
EAE	Experimental autoimmune encephalomyelitis
ER	Endoplasmic reticulum
ERK	Extracellular signal-regulated kinase
FITC	Fluorescein isothiocyanate
FST	Forced swim test
GABA	γ-aminobutyric acid
GFAP	Glial fibrillary acidic protein
GPx	Glutathione peroxidase
H&E	Hematoxylin and eosin
HD	Huntington’s disease
HEK	Human embryonic kidney
HO-1	Heme oxygenase-1
I_2_	Imidazoline I_2_
ICAM-1	Intercellular adhesion molecule-1
IFN-γ	Interferon-γ
IL	Interleukin
LAMP-2	Lysosome-associated membrane protein 2
LC	Locus coeruleus
LC3-II	Microtubule-associated protein 1 light chain 3-II
L-DOPA	L-3,4-dihydroxyphenylalanine
MAO	Monoamine oxidase
MAMs	Mitochondria-associated membranes
MCAO	Middle cerebral artery occlusion
MDA	Malondialdehyde
MLKL	Mixed lineage kinase domain-like protein
MMP	Matrix metalloproteinase
MOG	Myelin oligodendrocyte glycoprotein
MPO	Myeloperoxidase
MS	Multiple sclerosis
mTOR	Mechanistic target of rapamycin
NeuN	Neuronal nuclear protein
NLRP3	NOD-like receptor family pyrin domain-containing 3
NMDAR	N-methyl-D-aspartate receptor
NOX	NADPH oxidase
NOX2	NADPH oxidase 2
NR1	NMDAR subunit 1
NR2A	NMDAR subunit 2A
NR2B	NMDAR subunit 2B
Nrf2	Nuclear factor erythroid 2-related factor 2
NVU	Neurovascular unit
OGD	Oxygen-glucose deprivation
p62	Sequestosome 1
p70S6K	70-kDa ribosomal protein S6 kinase
PD	Parkinson’s disease
PET	Positron emission tomography
PI	Propidium iodide
PTZ	Pentylenetetrazol
RIPK1	Receptor-interacting protein kinase 1
RIPK3	Receptor-interacting protein kinase 3
RoB	Risk of bias
ROS	Reactive oxygen species
rt-PA	Recombinant tissue plasminogen activator
SCI	Spinal cord injury
SOD	Superoxide dismutase
SQSTM1	Sequestosome 1
SYRCLE	Systematic Review Centre for Laboratory animal Experimentation
TBI	Traumatic brain injury
TGF-β	Transforming growth factor-β
Th17	T helper 17 cell
tMCAO	Transient middle cerebral artery occlusion
TNF-α	Tumor necrosis factor-α
Treg	Regulatory T cell
TST	Tail suspension test
TUNEL	Terminal deoxynucleotidyl transferase dUTP nick-end labeling
vWF	von Willebrand factor
ZO-1	Zonula occludens-1
