# Supplementary material for: Therapeutic Potential of 2-(2-Benzofuranyl)-2-Imidazoline in Preclinical CNS Models: A Systematic Review of Mechanisms, Disease Models, and Cellular Targets
Source: Pharmaceuticals (Basel). 2026 Jul 24;19(8):1155. doi: 10.3390/ph19081155 (PMC13516425; doi:10.3390/ph19081155)
Supplement: Supplementary file 1 [file pharmaceuticals-19-01155-s001.zip › Supplementary Table S1.pdf]

Supplementary Table S1. Database-specific search strategies and records retrieved.

Searches were last updated on May 19, 2026. The final record set for each database was generated by combining query set #1 and query set #2. Records retrieved indicate the number of records identified before duplicate removal.

Database	Search set	Search concept	Search field / scope	Database-specific query	Records retrieved
PubMed	#1	2-BFI AND (brain OR neuro*)	MeSH Terms / All Fields	(("2 2 benzofuranyl 2 imidazoline"[Supplementary Concept]) OR ("2 2 benzofuranyl 2 imidazoline"[All Fields]) OR ("2 bfi"[All Fields])) AND (("brain"[MeSH Terms]) OR ("brain"[All Fields]) OR ("brains"[All Fields]) OR ("brain s"[All Fields]) OR ("neuro*"[All Fields]))	112
PubMed	#2	2-BFI AND (inhibit OR protect OR neuroprotect)	MeSH Terms / All Fields	(("2 2 benzofuranyl 2 imidazoline"[Supplementary Concept]) OR ("2 2 benzofuranyl 2 imidazoline"[All Fields]) OR ("2 bfi"[All Fields])) AND (("inhibit"[All Fields]) OR ("inhibition"[All Fields]) OR ("inhibits"[All Fields]) OR ("inhibited"[All Fields]) OR ("inhibiting"[All Fields]) OR ("protect"[All Fields]) OR ("protected"[All Fields]) OR ("protecting"[All Fields]) OR ("protection"[All Fields]) OR ("protective"[All Fields]) OR ("protective agents"[MeSH Terms]) OR ("neuroprotect"[All Fields]) OR ("neuroprotection"[MeSH Terms]) OR ("neuroprotection"[All Fields]) OR ("neuroprotective"[All Fields]) OR ("neuroprotective agents"[MeSH Terms]))	71
PubMed	#3	#1 AND #2	Combined search set	#1 AND #2	58
Web of Science	#1	2-BFI AND (brain OR neuro*)	All Fields	2-BFI AND (brain OR neuro*)	120
Web of Science	#2	2-BFI AND (inhibit OR protect OR neuroprotect)	All Fields	2-BFI AND (inhibit OR protect OR neuroprotect)	42
Web of Science	#3	#1 AND #2	Combined search set	#1 AND #2	39
Embase	#1	2-BFI AND (brain OR neuro*)	All Fields	'2-bfi' AND ('brain'/exp OR brain OR neuro*)	110
Embase	#2	2-BFI AND (inhibit OR protect OR neuroprotect)	All Fields	'2-bfi' AND (inhibit OR protect OR neuroprotect)	15
Embase	#3	#1 AND #2	Combined search set	'2-bfi' AND ('brain'/exp OR brain OR neuro*) AND '2-bfi' AND (inhibit OR protect OR neuroprotect)	10

Database	Search set	Search concept	Search field / scope	Database-specific query	Records retrieved
Scopus	#1	2-BFI AND (brain OR neuro)	Article title, abstract, keywords	TITLE-ABS-KEY (2-BFI AND (brain OR neuro))	64
Scopus	#2	2-BFI AND (inhibition OR protect OR neuroprotection)	Article title, abstract, keywords	TITLE-ABS-KEY (2-BFI AND (inhibition OR protect OR neuroprotection))	53
Scopus	#3	#1 AND #2	Combined search set	#1 AND #2	32
