# Supplementary material for: Therapeutic Potential of 2-(2-Benzofuranyl)-2-Imidazoline in Preclinical CNS Models: A Systematic Review of Mechanisms, Disease Models, and Cellular Targets
Source: Pharmaceuticals (Basel). 2026 Jul 24;19(8):1155. doi: 10.3390/ph19081155 (PMC13516425; doi:10.3390/ph19081155)
Supplement: Supplementary file 1 [file pharmaceuticals-19-01155-s001.zip › Supplementary Table S2A.pdf]

Supplementary Table S2A. Category-level summary of excluded reports.

Exclusion category	N
Unrelated or false-positive records	5
Review/background articles without eligible original data	3
Binding/radioligand/receptor or MAO-related characterization only	10
Non-CNS or peripheral pharmacology studies	4
CNS neurochemical/electrophysiological/pharmacodynamic studies without eligible disease-related protective or functional outcomes	5
Behavioral/pharmacological studies not meeting the predefined inclusion criteria	7
Overlapping or non-independent reports not counted as separate studies	1
Total excluded	35
